# Primary Electrical Heart Disease—Principles of Pathophysiology and Genetics

**DOI:** 10.3390/ijms25031826

**Published:** 2024-02-02

**Authors:** Krzysztof Badura, Dominika Buławska, Bartłomiej Dąbek, Alicja Witkowska, Wiktoria Lisińska, Ewa Radzioch, Sylwia Skwira, Ewelina Młynarska, Jacek Rysz, Beata Franczyk

**Affiliations:** 1 Department of Nephrocardiology, Medical University of Lodz, Ul. Zeromskiego 113, 90-549 Lodz, Polandsylwia.skwira@stud.umed.lodz.pl (S.S.);; 2 Department of Nephrology, Hypertension and Family Medicine, Medical University of Lodz, Ul. Zeromskiego 113, 90-549 Lodz, Poland

**Keywords:** channelopathy, arrhythmia, sudden cardiac death, long QT syndrome, Brugada syndrome, early repolarization syndrome, CPVT

## Abstract

Primary electrical heart diseases, often considered channelopathies, are inherited genetic abnormalities of cardiomyocyte electrical behavior carrying the risk of malignant arrhythmias leading to sudden cardiac death (SCD). Approximately 54% of sudden, unexpected deaths in individuals under the age of 35 do not exhibit signs of structural heart disease during autopsy, suggesting the potential significance of channelopathies in this group of age. Channelopathies constitute a highly heterogenous group comprising various diseases such as long QT syndrome (LQTS), short QT syndrome (SQTS), idiopathic ventricular fibrillation (IVF), Brugada syndrome (BrS), catecholaminergic polymorphic ventricular tachycardia (CPVT), and early repolarization syndromes (ERS). Although new advances in the diagnostic process of channelopathies have been made, the link between a disease and sudden cardiac death remains not fully explained. Evolving data in electrophysiology and genetic testing suggest previously described diseases as complex with multiple underlying genes and a high variety of factors associated with SCD in channelopathies. This review summarizes available, well-established information about channelopathy pathogenesis, genetic basics, and molecular aspects relative to principles of the pathophysiology of arrhythmia. In addition, general information about diagnostic approaches and management is presented. Analyzing principles of channelopathies and their underlying causes improves the understanding of genetic and molecular basics that may assist general research and improve SCD prevention.

## 1. Introduction

Channelopathies, also called primary electrical disorders of the heart, constitute a heterogenous cluster of diseases based on the disbalances of the ion currents without any structural cardiac defects [1]. They might stem from genetic polymorphisms and/or environmental factors, leading to the gain- or loss-of-function mutations in calcium (Ca^2+^), sodium (Na^+^), potassium (K^+^), and transient receptor potential (TRP) channels or genes controlling ion channel functions in cardiomyocytes [2]. These channels exhibit unique ion selectivity, playing a crucial role in the accurate and timely control of charged ion movement across the cell membrane in myocytes. The combined effect of their activity in cardiac muscle shapes the surface electrocardiogram [3].

The mutations predispose to lethal ventricular tachyarrhythmias and result in an elevated incidence of sudden cardiac death (SCD), also defined as sudden cardiac death syndrome (SCDS) and sudden infant death syndrome (SIDS) cases [4,5]. In accordance with the European Society of Cardiology (ESC) 2022 guidelines, SCD is an unexpected natural death believed to result from a cardiac origin [6]. It happens suddenly within one hour of the onset of symptoms in cases where there are witnesses and within 24 h of the last confirmed sighting when the incident is unwitnessed. In cases subjected to autopsy, SCD is characterized as an unforeseen natural death with an unknown or cardiac cause [6]. Moreover, SIDS is an unaccounted sudden death transpiring in an individual under the age of 1 year, with a negative evaluation in terms of pathology, toxicology, and forensic examination of the circumstances leading to the demise [6]. Both SCD and SIDS represent a global public health concern. SCD impacts millions of people worldwide and is estimated to account for 15–20% of total fatalities yearly [7]. At the same time, up to 35% of SIDS cases might be attributable to the frequently overlooked cardiomyopathies, ion channelopathies, or metabolic disorders [8]. Both SCD and SIDS definitions are presented in Table 1. According to available data, approximately 2% to 54% of sudden unexpected deaths in individuals under the age of 35 do not exhibit signs of structural cardiac abnormalities during autopsy [9]. This suggests that ion channelopathies are likely responsible for such cases [9].

The primary electrical heart diseases characterized by the lack of structural cardiac abnormalities comprise entities such as early repolarization syndrome (ERS), short QT syndrome (SQTS), long QT syndrome (LQTS), Brugada syndrome (BrS), idiopathic ventricular fibrillation (IVF), catecholaminergic polymorphic ventricular tachycardia (CPVT) [2].

Given the information available, we conducted an analysis based on scientific findings to facilitate a better understanding of the epidemiology, pathophysiology, diagnosis, and treatment of cardiac channelopathies.

## 2. Cardiac Arrhythmias in Primary Electrical Disease—General Aspects

As a primary electrical disease predisposed to SCD caused by ventricular arrhythmia (VA), the understanding of mechanisms leading to heart rhythm disorders seems to be crucial for a proper diagnostic approach and treatment. It is noteworthy that the majority of SCD in children, though it cannot be explained in autopsy, is thought to be caused by VA [7]. Among patients with primary electrical disease, often lethal VA includes ventricular fibrillation (VF), torsade de pointes (TdP), or ventricular tachycardia (VT) and its subtypes [7]. Cardiac arrhythmia may be simply defined as a non-physiological variation from normal heart rate and/or rhythm. In the following section, we discuss hypothetical mechanisms that may explain VA occurrence in structurally normal hearts. It should be noted that several pathophysiological mechanisms leading to VA remain unclear; however, underlying causes of VA have been widely studied in recent years.

### 2.1. Basic Mechanisms of Ventricular Arrhythmia

**Figure 1:**
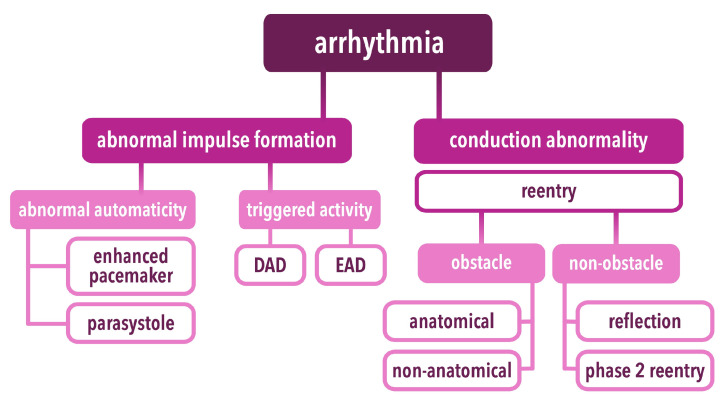
Pathophysiological mechanisms leading to arrhythmia. DAD—delayed afterdepolarization; EAD—early afterdepolarization.

In recent years, several pathophysiological mechanisms leading to cardiac arrhythmia have been proposed. These mechanisms are commonly divided into two groups: abnormal impulse formation and conduction abnormalities (Figure 1) [10]. It should be noted that mechanisms of both groups may occur and be complementary to one another, subsequently leading to arrhythmia [10,11,12]. According to patients with channelopathies who are at high risk of VA occurrence, both abnormal impulse formation and conduction disorders have an important role in initiating and maintaining life-threatening rhythm disorders [10,11,12].

#### 2.1.1. Abnormal Impulse Formation—Abnormal Automaticity

In normal conditions, cells of the electrical conduction system of the heart generate spontaneous action potential (AP) [10,13]. In phase 4 of the AP, during diastole, an inward current increases membrane potential, leading to the spontaneous depolarization of conduction cells [10,13]. The highest intrinsic rate is generated in the sinoatrial node (SAN); however, subsidiary structures of the conduction system as an atrioventricular node (AVN) and a bundle of His or Purkinje fibers also have an ability to generate AP, often with lower intrinsic rate when compared to SAN in physiological conditions [10]. Apart from physiological function, the intrinsic spontaneous activity of cardiomyocytes may be associated with abnormal impulse formation, often called abnormal automaticity, leading to rhythm disorders [10]. It should be noted that abnormal automaticity refers to the structures of the conduction system as well as other cardiomyocytes. Thus, the subdivision of abnormal automaticity has been proposed. Based on the dysfunctional structure, automaticity disorders are subdivided into two sub-disorders: pacemaker disorders and parasystole [10,14,15]. 

According to available data, pacemaker disorders seem to have a marginal role in life-threatening VA among patients with channelopathies. In humans, automaticity is a primary function of SAN, responsible for generating intrinsic impulses that are conducted through the heart, whereas SAN is considered the physiological pacemaker [10,16]. SAN cells have the shortest depolarization phase of the AP when compared to other subsidiary pacemakers, resulting in the highest rate of generated impulses that suppress other pacemakers [10,16]. It is noteworthy that 1 of 600 patients above the age of 65 suffering from CVD have SAN dysfunction [16]. Even though this article summarizes the available information about primary electrical heart diseases that often result in SCD due to the lethal VA, it should be noted that channelopathies may also occur in cells of the cardiac conduction system [16,17]. Interestingly, several studies indicate the role of mutations in genes encoding ion channels in SAN, AVN, and Purkinje fibers in dysfunction, presented as sinus pauses, bradycardia, or an atrioventricular block (AVB) [16,17]. Wallace et al. [16] reviewed molecular mechanisms and found that several genes involved in the development of SAN dysfunction are claimed to be the basis of well-established channelopathies leading to VA and subsequent SCD. Among the identified genes causing SAN dysfunction, as well as electrical disorders leading to VA, were *CASQ2*, *RyR2*, *KCNJ5*, *SLC8A1*, *SCN5A*, *HCN4*, and *ANK2* [16]. These observations are mainly based on a few case reports and animal studies, whereas the exact prevalence of these mutations among patients with SAN dysfunction remains unknown. Thus, patients with channelopathies seem to be at risk of automaticity disorders, especially SAN dysfunction; however, poor data quality and a lack of human studies indicate the need for further research [16,17]. 

According to the electrophysiological principles of heart rhythm disorders, premature ventricular complex (PVC), especially short-coupled PVC with cooccurring myocardial substrate, may initiate VA [10,18,19]. A short-coupled PVC may be defined as a PVC interrupting T-wave associated with a previous QRS complex [6]. Do et al. [15] analyzed the association of ventricular parasystole with VF and VT occurrence and showed there is a significant association in the group of patients with cardiomyopathies. To the authors’ knowledge, there is no evidence supporting the hypothesis that parasystole may induce VA in patients with channelopathies, indicating the need for further research.

#### 2.1.2. Abnormal Impulse Formation—Afterdepolarization and Triggered Activity

**Figure 2:**
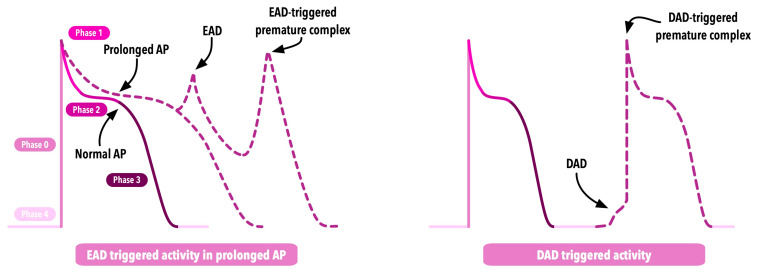
EAD and DAD formation. Phases of action potential are presented in boxes. AP—action potential; DAD—delayed afterdepolarization; EAD—early afterdepolarization.

Afterdepolarizations play an important role in arrhythmogenesis and are responsible for various heart rhythm disorders comprising atrial and ventricular arrhythmias. They are divided into two groups—early afterdepolarizations (EADs) and delayed afterdepolarizations (DADs) [20]. The division is based on the period of the AP of cardiomyocyte in which afterdepolarization occurs [20]. EADs occur during the repolarization, whereas DADs occur after the repolarization is complete [20]. Both DAD and EAD are visualized in Figure 2.

##### Early Afterdepolarization (EAD)

EADs often occur in states associated with a prolonged duration of AP repolarization that plays an important role in EAD formation and triggered activity pathogenesis. There are several theories explaining mechanisms leading to EADs; however, experimental and computational data remain inconclusive and indicate the need for further research. In the pathogenesis of EAD among patients with channelopathies, the role of inward currents secondary to prolonged repolarization should be emphasized [20]. There are two major currents that are claimed to have a leading role in EAD formation—I_CaL_, an L-type Ca^2+^, and I_NCX_ current generated by the Na^+^-Ca^2+^ exchanger. I_CaL_ has an important role in EAD formation; however, this current, in normal conditions, reduces during repolarization [20]. Prolonged repolarization may lead to the partial recovery of I_CaL_, secondary to L-type Ca^2+^ channel recovery caused by decreased intercellular Ca^2+^, due to the uptake by the smooth endoplasmic reticulum (SER) and Na^+^-Ca^2+^ exchanger activity [20,21]. Moreover, depolarized membranes may influence the voltage-dependent inactivation of I_CaL_ [20]. When combined, these two major mechanisms lead to the cooccurrence of opened and inactivated L-type Ca^2+^ channels in the plateau phase, whereas partially recovered I_CaL_ may lead to depolarization, causing EAD [20]. The reduction of an outward K^+^ current in phases 2 and 3 of AP that occurs in LQTS increases the role of I_CaL_ in depolarization due to the absence of outward current-inhibiting depolarization [20]. Without outwards currents predominating, I_CaL_ causes EAD by reaching the threshold for afterdepolarization [20]. Moreover, during phases 2 and 3 of AP, an I_NCX_ current generated by the Na^+^-Ca^2+^ exchanger increases Ca^2+^ levels within cardiomyocytes. Prolonged phases 2 and 3 of AP may result in an additional increase of cytoplasmatic Ca^2+^ that leads to EAD [20,21]

It is claimed that EADs are highly arrhythmogenic in patients with genetic or acquired long QT syndromes [21,22,23]. In recent years, it has been observed that EAD, often revealed in electrocardiogram (ECG) recordings as an R-on-T phenomenon, may initiate TdP, which is a polymorphic VT with a high risk of degeneration into VF and subsequently to SCD [24,25]. Interestingly, commonly presented in ECG, R-on-T may have two different underlying mechanisms: R-to-T and R-from-T [26]. The R-to-T phenomenon describes a situation when PVC encounters a refractory region and subsequently leads to a unidirectional conduction block and reentry development [26]. In this case, R and T waves are considered as independent events [26]. In the R-from-T mechanism, PVC emerges from the T-wave in the repolarizing region with further unidirectional propagation away from repolarization, which is called unidirectional propagation [26]. The R-from-T mechanism can cause repeated PVCs as well as evolve into reentry causing focal and re-entrant arrhythmias, respectively [26]. It is claimed that the R-to-T mechanism plays a major role in arrhythmogenesis in general cardiac diseases, whereas the R-from-T mechanism is rather associated with arrhythmia occurrence in patients with LQTS [26]. Alexander et al. [27] showed that in LQTS conditions, PVC that initiates TdP is not directly associated with EAD, whereas a steep voltage gradient at the border of long AP duration myocardial islands is claimed to be more relevant in VA formation [27]. Moreover, the results of this study indicate the important mediating role of I_CaL_ in PVC initiation, with the aim for low doses of calcium canal blocker therapies to abolish VA in LQTS patients [27]. EADs occurring near the border of long AP duration islands caused the increase in local voltage gradients, emphasizing the role of EADs in TdP induction. Steep gradients were only partially caused by EADs [27]. 

PVC has a primary role in inducing TdP, whereas the mechanism of perpetuation of arrhythmia remains not fully explored [22,24,25]. Some researchers indicate that TdP requires two factors: PVC secondary to prolonged AP and coexisting substrate in the form of repolarization heterogeneity [23]. The perpetuation mechanism of TdP can be divided into three groups: focal activity perpetuated TdP, reentry perpetuated TdP, and mixed perpetuation TdP [28]. The study of Vandersickel et al. [28] indicates that longer-lasting TdP (above 14 beats) is commonly perpetuated by the reentry mechanism, whereas shorter TdPs are predominantly maintained by the focal mechanism. The authors of this study observed that TdP based on the reentry mechanism tends to have shorter inter-beat intervals when compared to focal activity-perpetuated TdP [28]. Moreover, it was observed that focal perpetuated TdP tends to terminate spontaneously [28]. It is claimed that the decrease in the inter-beat interval observed in ECG recordings in humans with TdP may suggest a reentry mechanism [28]. 

In rare cases, TdP may occur with normal QT interval duration due to short-coupled PVC with coupling intervals under 400 ms (some authors suggest 300 ms) [29,30]. To describe this condition, the term “short-coupled TdP” was proposed [29]. Due to the low incidence, the molecular basis leading to scTdP remains unclear and requires further investigation. A systematic review by Wang et al. [29] included 103 patients with diagnosed scTdP from 22 studies and showed that PVC predominantly originates in Purkinje fibers and RVOT. Interestingly, scTdP originating from Purkinje fibers is more likely to degenerate into VF [29]. To date, the *DPP6* and *RyR2* variants are claimed to contribute to scTdP formation [31]. Genetic analysis conducted by Touat-Hamici et al. [32] showed that *RYR2* variant I748F in SPRY1 may be pathogenic, changing the thermal stability of the SPRY1 domain, leading to spontaneous Ca^2+^ release and further VA. Other *RYR2* variants reported to be associated with scTdP are *RyR2*-V1024I, *RyR*-N1151S, *RyR2*-S4938F, *RyR2*-M995V, and *RyR2*-H29D [33,34,35]. Finally, Sonoda et al. [36] identified an *SCN5A* mutation in a patient with scTdP. It should be noted that all variants above require further assessment, whereas currently available data suggest a potential genetic basis for scTdP. 

##### Delayed Afterdepolarization (DAD)

Similarly, DADs are often associated with an increased level of Ca^2+^ inside cardiomyocytes due to Ca^2+^ overload of the SER and/or decreased function of the Na^+^-Ca^2+^ exchanger that is responsible for a Ca^2+^ outflow from cardiomyocytes [20]. During sympathetic stimulation, enhanced I_CaL_, accompanied by increased protein kinase A (PKA) and CaMKII, leads to increased cytoplasmic Ca^2+^, with subsequent activation of ion channels contributing to the inward flow of Ca^2+^ into the SER to maintain the diastolic level of Ca^2+^ [20,37]. The inability to store Ca^2+^ due to its overload causes the subsequent leakage of Ca^2+^ back from the SER to the cytoplasm among channels associated with Rianodine receptors 2 (RyR2), which occurs after complete repolarization [20]. Moreover, especially in channelopathies such as CPVT, the abnormal function of RyR2 causes profuse Ca^2+^ leakage during diastole with the absence of I_CaL_ [37]. Spontaneous cytoplasmatic Ca^2+^ increase during diastole leads to the overactivation of the Na^+^-Ca^2+^ exchanger that generates an inward current, causing depolarization called DAD and subsequent AP in a mechanism of triggered activity [37]. 

DAD-triggered activity underlies several supraventricular (SVT) and VT types, such as focal atrial tachycardias, AF accelerated junctional rhythms, exercise-induced VT, or VT secondary to digoxin toxicity [20,38,39,40,41]. In channelopathies, DAD plays a primary role in CPVT formation (see Section 3.6).

#### 2.1.3. Reentry

Abnormal impulse conduction, where an impulse fails to extinguish and subsequently activates a region closely after the refractory period, is called reentry [42]. Reentry can be divided into two types based on the mechanism of its formation: circus-type associated with an obstacle and non-obstacle reentry comprising reflective and phase two reentry types [10,42].

##### Obstacle Reentry (Circus-Type)

Circus-type reentry is caused by an anatomical and/or physiological obstacle. The impulse cannot be conducted through the obstacle, whereas it circulates around the nonconductive region that causes a reentry circuit [42]. In the year 1913, Mines [43] formed three following principles: (i) an area of unidirectional block must exist; (ii) the excitatory wave must propagate along a distinct pathway and return to its point of origin, then it starts again; (iii) the interaction of the circuit at any point should terminate the circus movement. Presented principles still remain actual and explain the general mechanism of circus-type reentry. Typically, an anatomical obstacle causing reentry tachycardia occurs in atrio-ventricular nodal reentry tachycardia (AVNRT), atrio-ventricular reentry tachycardia (AVRT), and subsequently, to the myocardial scar occurrence due to myocardial ischemia or injury [10,11,44,45,46]. In patients without structural heart disease, the functional obstacle reentry mechanism seems to be more relevant than anatomical obstacle reentry in malignant VA pathogenesis. Functional obstacle reentry is highly associated with premature contractions caused by abnormal impulse formation (see Section 2.1.1) [10,42,47]. Although the pathophysiology of functional obstacle reentry remains unclear, there are a few theories trying to explain this phenomenon. In the series of publications, Alessie et al. [47,48] presented the concept of functional reentry based on the rabbit’s atrium model. The results of this study indicate that premature stimulation of atrial tissue may cause tachycardia [47]. Later, this phenomenon was named the “leading cycle” [48]. The premature impulse is conducted through areas with decreased refractory time, resulting in the formation of areas with blocked conduction, with the impulse circulating around [10,47,48]. According to Alessie et al. [47,48], the theory suggests that the central region is activated faster than the impulse circulates due to the “centripetal wavelets”. Another theory to explain this phenomenon, called the “spiral wave”, assumes that an ectopic impulse causes the wavefront crossing another front in the refractory period of the previous beat of the leading rhythm [49]. Apart from the pathophysiology, it should be noted that leading cycle theory and spiral wave theory have, in their origins, a premature contraction that may be caused by abnormal impulse formation. This phenomenon often occurs in patients with channelopathies, with a greater incidence [20,47,48,49,50,51,52].

##### Non-Obstacle Reentry

Non-obstacle reentry occurs without circus impulse conduction and may be subdivided into two subgroups: reflection reentry and phase 2 reentry [10,42]. 

Reflection reentry remains not fully explored; however, current explanation approaches divide reflection reentry into two types: segment-type reflection and expansion-type reflection [42]. Segment-type reflection is based on a three-region model comprising the proximal and distal regions divided from each other by an area of decreased conduction [42,53,54]. Decreased conduction from the proximal to distal region causes a delay in the activation of the distal region that may subsequently lead to the retrograde conduction of the impulse [42,53,54]. The repolarized proximal region is prone to retrograde activation caused by the recurrent impulse from the distal region [42,53,54]. Expansion-type reflection reentry assumes the presence of an isthmus between Purkinje fibers, where an impulse is conducted from the narrow part to the expanded region, activating a greater number of cells that predisposes an impulse to return to the narrow part, forming a loop of reentry [42,53,54]. 

Phase 2 reentry is caused by abnormalities in the AP, especially in epicardial myocytes. Epicardial myocytes typically have a spike-shaped phase 1 of the AP and a dome-shaped phase 2, as well as the shortening of phase 2 of the AP predisposes to VA [55]. In normal conditions, at the end of phase 1 of the AP, mainly two currents, I_to_ and I_Na_, occur and lead to a transmembrane balance causing an AP notch [55,56]. Then, the AP notch is followed by the dome-shaped phase 2 of the AP; meanwhile, at the beginning of phase 2, an I_CaL_ current dominates [55,57]. The inhibition of I_Na_ and/or I_CaL_ with maintained or enhanced I_to_ results in the absence of a dome shape and shortening of phase 2 [57,58]. Apart from the currents mentioned above, other currents such as I_Kr_, I_Ks_, I_K-ATP_, and I_Cl_, when enhanced, may predispose to phase 2 reentry; however, these seem to have a minor role in reentry pathogenesis [57]. The shortening of phase 2 of the AP alone is not sufficient to form reentry, whereas accompanied by the region of normal AP, it forms a substrate for malignant arrhythmias [55,57]. The impulse is conducted from depolarized cells during the dome-shaped phase 2 into already repolarized cells with shortened AP due to the abnormal dome-shaped phase 2 [55]. Then, the improper reactivation of abnormal phase 2 cells occurs, forming a reentry called phase 2 reentry, leading to the pathological state called the dispersion of repolarization [55,56]. A mathematical modeling study conducted by Maoz et al. [58] showed that formation of phase 2 reentry may be rather associated with ongoing changes in AP morphology due to the imbalance in certain groups of cells, not a constant AP morphology occurring in several cells. Therefore, it should be noted that alternations of phase 2 of the AP may vary over time in each cardiomyocyte and change from normal (dome-shaped pattern) to abnormal (shortened pattern) [58].

The clinical significance of phase 2 reentry is present in patients with BrS, where two hypotheses of its pathogenesis were presented [55,57]. The phase 2 reentry in repolarization hypothesis in BrS explains the malignant VA occurrence (see Section 3.3). Interestingly, Koncz et al. [56] assessed transmembrane AP in a model of the canine ventricle and showed that ERS is caused by a preferential accentuation of the AP notch. The transmural gradient across the walls of ventricles, associated with a notch in AP, can be present in both left and right ventricles, causing ERS and BrS ECG patterns, respectively [56]. In ERS, an increased function of I_K-ATP_ and decreased I_CaL_ and/or I_Na_ leads to the dispersion of repolarization with a risk of VT/VF occurrence [56]. It should be noted that Koncz et al. [56] proposed an explanation for the increased risk of VT/VF occurrence in patients with ERS patterns revealed in inferior leads of ECG (II, III, aVF) [59,60]. The study showed that the higher activity of I_to_ occurs in the epicardium associated with the inferior wall of LV when compared to the lateral wall [56].

### 2.2. Multifactorial Mechanism Underlying Arrhythmia in Channelopathies

Although it seems well-established that EAD-associated PVCs have an important role in inducing VA among patients with LQTS, in contrast, DADs in CPVT have a primary role; in IVF, ERS, and SQTS, the mechanism leading to arrhythmia is more composite. 

In IVF, mutations occur in the following genes: *CALM1*; *IRX3*, encoding Iroquois homeobox gene family transcriptor factor; *RYR2,* and a promoter haplotype in the *DPP6* gene locus on chromosome 7. The *CALM1* mutation is associated with dysregulated binding of calmodulin to channels, leading to enhanced calcium-dependent gating, channel assembly with accompanying impaired conduction–contraction coupling, and pathological refractory periods [61]. In patients with *CALM1* mutation, the ECG may reveal prolonged QTc, whereas QTc prolongation and electrical instability are associated with impaired function of Ca^2+^-dependent channel function [61]. Other mutations are associated with PVC-induced VF or TdP [61]. The *IRX3* mutation leads to the increased transcription of *SCN5A* and connexin-40 genes, resulting in cardiac conduction system disturbances [61]. *DPP6* promoter mutation leads to increased *DPP6* expression and results in enhanced inactivation of I_to_ [61]. It is noteworthy that several patients with IVF had spontaneous PVCs originating in the conduction system or repetitive activity caused by programmed electrical stimulation [62]. Observed activity comprised VT with further degeneration into VF; however, reentry in the left bundle branch was excluded [62]. Moreover, an electrophysiological study of patients with IVF often reveals regions of enhanced conduction without any structural abnormalities [62]. 

In SQTS, mutations of genes encoding both K^+^ and Ca^2+^ channels may occur. The gain-of-function mutations of genes encoding K^+^ (see Section 3.2) channels leads to altering repolarization that manifests in the ECG as a short QTc. Creating increased peak current density accompanied by a positive shift of steady-state inactivation subsequently constitutes a substrate for reentry and VF [63]. In the case of Ca^2+^ channel loss-of-function, inhibited I_CaL_ leads to QT interval shortening [64]. Some authors compare the augmented function of K^+^ channels and the inhibited function of Ca^2+^ channels to hyperkalemia and hypocalcemia, respectively [64].

## 3. Primary Electrical Cardiac Diseases

### 3.1. Long QT Syndrome (LQTS)

#### 3.1.1. Epidemiology

In 2009, Schwartz et al. [65] conducted the inaugural prospective study involving 44,596 infants, combining ECG recordings with genetic analysis of seven LQTS genes in individuals with established prolonged QTc > 470 ms. The study revealed a prevalence of congenital LQTS of at least 1:2534 apparently healthy live births [65]. Notably, the study underscored that, in contrast to other channelopathies, congenital LQTS is more commonly overlooked than considered rare [65]. The prevalence may even be higher, as molecular screening was limited to infants with a QTc > 470 ms, suggesting a real prevalence of around 1:2000 [65]. Much earlier, Schwartz et al. hypothesized that the prevalence of LQTS, when accounting for silent carriers (genotype-positive, phenotype-negative individuals), was substantially greater [65]. Data from the Exome Sequencing Project in 2013 indicated a prevalence of the “pathogenic” LQTS genotype to be 1:80, a significant discordance with the expressed QTc clinical phenotype prevalence of 1:2000 [66]. Even when considering incomplete penetrance and variable expressivity of LQTS genes, the basis for this discrepancy remains incompletely elucidated [66].

#### 3.1.2. Genetic and Molecular Aspects

The QT interval is the period between the start of ventricular depolarization and the conclusion of ventricular repolarization. Because abnormalities in the genes that encode cardiac ion channels result in the prolongation of phase 1 of the AP in the majority of congenital LQTS patients, congenital LQTS is classified as a cardiac channelopathy [66]. Congenital LQTS is a pathological condition characterized by an aberration in the repolarization of the myocardium, as indicated by an extended QT interval observed on ECG [67]. This anomaly can precipitate VA and ultimately culminate in an abrupt demise of the cardiac system [67]. 

Depending on the category of monogenic mutation, long QT syndrome is presently categorized into 17 subtypes, wherein LQT1, LQT2, and LQT3 are the prevailing manifestations. Only three genes, namely *KCNQ1*, *KCNH2*, and *SCN5A*, were systematically documented as definitive contributors to typical long QT syndrome (LQTS), with each gene corresponding to major forms of LQTS denoted as LQT1, LQT2, and LQT3, respectively. Four additional genes, namely *CALM1*, *CALM2*, *CALM3*, and *TRDN*, exhibit compelling or conclusive evidence implicating them in LQTS etiology, albeit with atypical manifestations [68]. What is more, mutations in the *CACNA1C* gene, a voltage-gated calcium channel gene, are integral to the complex syndrome known as Timothy syndrome (LQT8), which is characterized by a highly malignant form of LQTS frequently presenting with a 2:1 functional AVB and multi-systemic disorders. It represents a rare variant of congenital LQTS. Notably, mutations in *CALM1-3* genes are currently recognized as calmodulinopathy, while mutations in the *TRDN* gene are designated as Triadin Knockout Syndrome. In the context of LQTS, LQT1 and LQT2 involve loss-of-function mutations in the *KCNQ1* and *KCNH2* genes, respectively, affecting potassium channels. These mutations result in decreased activity of the slow delayed rectifier current (I_Ks_) and rapid delayed rectifier current (I_Kr_) during phase 3 of the AP, respectively. Conversely, LQT3 is associated with a gain-of-function mutation in the *SCN5A* gene affecting the sodium channel during phase 0 of the AP, leading to persistent sodium influx, extending through the plateau phase. The impairment of I_Ks_ or I_Kr_ function, or the enhancement of I_Na_ function, generally predisposes ventricular myocytes to EADs. When the synchronous development of EADs occurs in myocardial regions, it has the potential to initiate life-threatening VA, including TdP [66].

#### 3.1.3. Diagnosis

Clinical symptoms of congenital LQTS vary widely, with the likelihood of malignant consequences being heavily influenced by the differences in molecular genetics in each variety [66]. Congenital LQTS clinical signs include syncope or convulsions, abortive cardiac arrest, and abrupt cardiac death. Many people with congenital LQTS will be asymptomatic their whole lives. According to Mayo Clinic data, just 27% of patients were symptomatic prior to their first clinical examination [69]. The median age at the onset of the first symptom was 12 years. LQTS-related syncope is arrhythmogenic in nature, with the genesis often being polymorphic VT. Syncopal episodes with tonic–clonic movements may be mistaken as epilepsy. The majority of arrhythmias seen in patients with congenital LQTS are ventricular tachyarrhythmias, with polymorphic VT being the most common. Atrioventricular (AV) block, atrial arrhythmias, and the not-so-rare associated sinus bradycardia can occur in a minority of individuals with congenital LQTS [70]. The most prevalent related findings in LQTS patients are hearing loss and congenital heart disease. According to the Mayo Clinic data released by Rohatgi et al. [69], one out of every four previously symptomatic patients had at least one non-lethal LQTS trigger and cardiac episode. According to the same study, the mortality rate of congenital LQTS with effective medical management is now 0.3% [69].

The initial diagnostic assessment for congenital LQTS involves a comprehensive approach, encompassing detailed personal and multi-generational family history, physical examination, 12-lead ECG recordings, and the computation of the Schwartz score—a diagnostic metric detailed in Table 2. This initial evaluation aims to exclude secondary causes of congenital LQTS, emphasizing the need to distinguish it from acquired LQTS. Patients deemed to have a high likelihood of congenital LQTS are advised to undergo additional assessments, including 24 h ambulatory monitoring, treadmill or cycle stress testing, and genetic testing for definitive confirmation. Noteworthy manifestations such as syncope, seizures, and sudden cardiac arrest are crucial aspects of personal history evaluation [70].

Family history inquiries focus on premature sudden deaths, unexplained accidents, drownings, or seizure disorders. Syncope episodes with seizure characteristics are particularly scrutinized, as they are frequently misdiagnosed as epilepsy within LQTS families.

Physical examination in most LQTS patients typically reveals no specific abnormalities, although certain patients may exhibit concurrent anomalies associated with specific LQTS syndromes. For instance, congenital deafness may indicate Jervell–Lange-Nielsen syndrome, while skeletal abnormalities such as short stature and scoliosis may be indicative of Andersen–Tawil syndrome. Additionally, congenital heart diseases, cognitive and behavioral problems, musculoskeletal diseases, and immune dysfunction may suggest Timothy syndrome [71].

The 12-lead ECG, with a focus on the corrected QT interval (QTc), remains a central diagnostic and prognostic parameter for LQTS. Despite some criticism, Bazett’s formula is commonly employed for QTc calculation. Abnormal T wave morphologies, including biphasic or notched patterns and T wave alternans, are common findings in congenital LQTS patients, with the latter serving as a marker of heightened electrical instability and a precursor to torsade de pointes [66].

Exercise testing, specifically exercise ECG stress tests, plays a valuable role in the diagnostic evaluation. It is particularly effective in triggering arrhythmias and detecting changes in T wave morphology and abnormal QT responses during recovery. Notably, patients with LQT1 may exhibit QT interval characteristics that differ from those with LQT2 or LQT3, highlighting the subtype-specific nature of exercise-induced responses [72]. 

The Schwartz score, proposed by Dr. Peter Schwartz, serves as a key diagnostic tool, with a high probability score indicating an approximately 80% chance of a positive LQTS genetic test. Intermediate probability scores warrant further investigation, while low scores (<1 point) discourage genetic testing [6].

Ambulatory ECG monitoring, such as Holter monitoring, is useful for detecting intermittent QT prolongation, bradyarrhythmia, macroscopic T wave alternans, and T wave notching, providing insights into dynamic changes throughout the day [73].

Genetic testing is increasingly recognized as a standard of care in LQTS diagnostic and prognostic evaluations, offering benefits in establishing uncertain diagnoses, identifying affected family members, and informing personalized treatment plans based on the specific causative mutation. The prognostic and therapeutic significance of genetic testing is particularly valuable, as certain mutations are associated with more malignant forms of the disease. Despite the complexity and heterogeneity of congenital LQTS, genetic testing remains integral to a comprehensive patient management strategy [66,72].

Current guidelines recommend genetic testing in cases of high or intermediate clinical suspicion based on history, family history, ECG findings, and Schwartz score [70]. Additionally, asymptomatic patients with specific QTc values and cascade/variant-specific testing for relatives with identified disease-causing variants are recommended. It is crucial to note that a negative genetic test does not definitively rule out congenital LQTS due to the existing complexities in its genetic etiology [70].

#### 3.1.4. Management

The current management of long QT syndrome (LQTS) lacks causal treatment. Treatment options include lifestyle modifications, medication therapy with a focus on beta-blockers, device therapy, and surgical interventions. Beta-blockers, serving as the first-line therapy, address the common trigger for cardiac events, primarily a sudden increase in sympathetic activity [66,72]. 

While antiadrenergic therapies, particularly beta-blockers, offer significant protection, not all cardiac events in LQTS result from sympathetic activation. Genetic factors play a role, with some patients experiencing events during sleep, rest, or sudden arousal. Beta-blockers, such as nadolol, are highly effective, especially in LQT1 patients, reducing cardiac event rates significantly [74]. 

For asymptomatic patients with QTc < 470 milliseconds, beta-blocker therapy may not always be necessary. Risk–benefit calculations may favor a non-therapy approach in certain cases, particularly in older patients with genetically lower-risk LQTS subtypes [74]. 

In cases where beta-blocker therapy is insufficient or intolerable, alternative treatments may include mexiletine therapy, left cardiac sympathetic denervation (LCSD), and implantable ICD placement. Mexiletine, particularly for LQT3 patients, has mutation-specific effects, and LCSD involves removing thoracic ganglia to interrupt norepinephrine release. ICDs play a crucial role, especially in cases of documented cardiac arrest or recurrent major events [75]. 

Lifestyle modifications, including avoiding medications with QT-prolonging potential, managing electrolyte balance during illnesses, and addressing fever, are recommended for all LQTS patients. Recreational activity is generally allowed, with professional athletes requiring evaluation by an LQTS specialist for competitive sports participation [76]. Concerns about β-blocker therapy efficacy in LQT2 patients were raised by a study led by Priori et al. The current research contradicts this by demonstrating a substantial 64% reduction in the risk of cardiac events in high-risk LQT2 patients, attributing higher residual event rates to an overall increased event rate in adolescent and adult patients rather than diminished therapy efficacy [77]. Another study by Vincent et al. [78] focused on β-blocker therapy compliance in LQT1 patients, showing significant benefits and a low residual event rate. In contrast, the present analysis, targeting LQT1 males during the high-risk childhood period, identified a higher residual event rate despite medical therapy. Importantly, non-compliance did not impact the results, reinforcing the efficacy of β-blocker therapy in the current study [76].

The study recommends routine β-blocker therapy for high-risk LQT1 and LQT2 patients and suggests primary ICD therapy if syncope occurs during treatment or when compliance/intolerance to β-blocker therapy is a concern. The findings highlight that LQTS patients experiencing syncope during β-blocker therapy have a relatively high subsequent rate of life-threatening events, emphasizing the need for vigilant management. ACA or SCD rarely occur as presenting symptoms in patients treated with β-blockers. The proposed strategy categorizes patients into low- and high-risk groups based on age, gender, syncope history, and QTc duration, tailoring therapeutic decisions accordingly. Limitations include a small subset of patients with SCD prior to genetic testing and potential bias in retrospective data collection [79]. 

Goldenberg et al. [76] propose a management strategy for LQT1 and LQT2 patients based on findings from the International LQTS Registry. The recommendations categorize patients into low- and high-risk groups:

- Low-risk LQT1 and LQT2 patients (including LQT1 females and LQT2 patients aged 0–14, and LQT1 patients and LQT2 males aged 15–50, without prior syncope and with a QTc < 500 ms) did not show a statistically significant benefit from β-blocker therapy in the study. Therefore, for low-risk patients, the recommendation is to consider β-blocker therapy on an individual basis and initiate routine medical therapy if symptoms arise or follow-up ECGs indicate an increase in QTc duration.
- High-risk LQT1 and LQT2 patients (including LQT1 males aged 0–14, LQT2 females aged 15–40, patients with a history of syncope and/or documented TdP without β-blocker therapy, and patients with a prolonged QTc) demonstrated a significant and pronounced reduction in the risk of cardiac events after the initiation of medical therapy. These high-risk patients are recommended for routine β-blocker therapy as a first-line measure without contraindications.
- Primary ICD therapy is recommended for high-risk LQT1 and LQT2 patients if syncope and/or TdP occur during medical therapy or if there are concerns about compliance or intolerance to β-blocker therapy.
- Secondary prevention with an ICD is advised for all LQTS patients who experience ACA.

In all cases, the choice of treatment should be tailored and based on the individual’s risk profile, the specific LQTS subtype, with a multidimensional approach involving medication, surgical interventions, and lifestyle adjustments [66,72].

### 3.2. Short QT Syndrome (SQTS)

#### 3.2.1. Epidemiology

Short-QT syndrome (SQTS) is diagnosed when a corrected QT (QTc) interval is shorter than 360 ms because of innate or acquired factors [80]. It belongs to the spectrum of early repolarization syndrome and was first described in literature in 2000 by Gussak et al. based on case reports of a 17-year-old Caucasian female and a 37-year-old Caucasian female. A 17-year-old patient experienced intraoperative AF with a rapid ventricular response of unknown etiology. Having examined her relatives, a 21-year-old brother (QT: 272 ms; HR: 58 beats per minute; BPM) and a 51-year-old mother (QT: 260 ms; HR: 74 BPM), it transpired that the family had SQTS. At the same time, the 37-year-old patient’s episode of syncope, initially of unidentified cause, was subsequently attributed to SQTS following evaluation [81]. It is difficult to estimate the accurate prevalence of SQTS because of the rarity of occurrence and diagnosis of this disease entity. To establish appropriate statistics, Funada et al. [82] analyzed the ECG of 10,984 Japanese patients. In total, 158 patients had QTc shorter than 354 ms, and only three patients presented with QTc < 300 ms. Nevertheless, none displayed clinical symptoms associated with short QT syndrome [82]. The prevalence of SQTS was also screened for in the Kherameh cohort study, one of the southern branches of the Prospective Epidemiological Research Studies in Iran (PERSIAN). Out of 4363 adult patients, approximately 72 patients (1.65%) had a mean QTc of 360.72  ±  11.72 ms. Also, a minimum of two individuals exhibited a high likelihood of SQTS, and three individuals with an intermediate probability of SQTS were identified [83]. Additionally, available data suggest that the prevalence of males in the short QT interval group points out that, typically, females tend to have a longer QT interval [84].

#### 3.2.2. Genetic and Molecular Aspects

SQTS represents a primary electrical disorder with low penetrance, following an autosomal dominant inheritance pattern [85]. SQTS can be attributed to the gain-of-function mutations in the K^+^ channel genes *KCNH2*, *KCNQ1*, and *KCNJ2,* which account for the SQT1-3 genotypes. The loss-of-function mutations in L-type Ca^2+^ channel subunits *CACNA1C, CACNB2,* and *CACNA2D1* are described as SQT4-6 genotypes. As well as a Na^+^ channel gene SQT7, The R689H mutation was discovered in *SCN5A*, and biophysical analysis revealed that the SCN5A protein containing this mutation could not facilitate I_Na_, indicating a loss-of-function [86,87,88]. The comparison of the aforementioned SQTS subtypes regarding genes, types of mutation, and subunits can be found in Table 3 [89]. Recently, the Solute Carrier Family 4 Member 3 (*SLC4A3*) anion exchanger Cl^−^/HCO_3_^−^ was recognized to contribute to SQTS and described as the 8th SQT genetic variation [90]. Its impact was established based on the animal model of zebrafish, as well as in two unrelated families with SQTS. Knocked-out *SLC4A3* zebrafish presented with increased intracellular pH, shortened QTc, and reduced systolic duration [90]. Christiansen et al. [91] conducted a study in which 5 out of 34 patients with SQTS had *SLC4A3* mutations, and new variants were examined: c.1798C > T (p.Arg600Cys), c.1861C > T (p.Arg621Trp), c.2556G > C (p.Glu852Asp), and c.2855G > A (p.Arg952His) [91]. 

#### 3.2.3. Diagnosis

The identification of SQTS relies on assessing the patient’s family history, evaluating symptoms, examining a 12-lead ECG, and ruling out secondary factors such as hypercalcemia, hyperkalemia, hyperthermia, acidosis, and alterations in autonomic tone. The ESC guidelines from 2022 recommend establishing a diagnosis based on the shortening of QTc and one or more of the following criteria: a confirmed pathogenic mutation, a family history of SQTS, or survival from a VF/VT episode in the absence of heart disease. Additionally, the possibility of SQTS diagnosis arises when a QTc ≤ 320 ms is present or when the QTc falls within the range of 320 to 360 ms, along with a history of arrhythmic syncope. Finally, SQTS diagnosis may be contemplated if the QTc ranges between 320 and 360 ms, and there is a family history of sudden death below the age of 40 years [6]. Moreover, Schwartz’s score may aid in categorizing the likelihood of SQTS, where a total score of ≤2 suggests a low probability, 3 indicates an intermediate probability, and a total score of ≥4 points to a high probability of SQTS [89].

What is also worth taking into consideration is the association of QT interval in SQTS, HR, and physical exercise tests. In the study by Giusetto et al. [92] performed on 21 SQTS patients, it was shown that patients exhibited a reduced adaptation of the QT interval as HR increased. The average variation in the QT interval from rest to peak effort was 48 ± 14 ms, in contrast to a mean of 120 ± 20 ms observed in the control group (*p* < 0.0001) [92].

Yet, the clinical presentations of SQTS remain unspecific. An analysis of case reports by Giustetto et al. [93] suggests that cardiac arrest is the first clinical presentation (in 28% of cases) and the most common symptom (34% cases).

Screening criteria for children and young adults using manually measured QTc were additionally established by Hazeki et al. A combined 75,040 Japanese adolescents participated in the study. Subsequent cut-offs were established for males: 1st graders: 325 ms, 7th graders: 315 ms, 10th graders: 305. This was also performed for 320 ms females in the 1st, 7th, and 10th grades [94]. It is worth emphasizing that implantable loop recorders can improve risk assessment of SQTS complications in young patients [95]. Despite SQTS being a rarely anticipated disease unit in adolescents, it is worth considering it in the differential diagnosis to prevent life-threatening arrhythmia.

Genetic testing seems to be a useful diagnostic tool in SQTS; nevertheless, its utility remains questionable, given that the genotype–phenotype association is not clearly defined. At the same time, there might be various genetic mutations yet to be discovered and are not covered in the common screening protocol. However, available data suggest that genetic testing might be conventional in family members of probands who died suddenly without apparent cause. Still, the implementation of prophylactic treatment should be considered only when combined with an assessment of the phenotype [96].

#### 3.2.4. Management

For symptomatic SQTS patients with a life expectancy exceeding 30 years and at a risk of SCD, an implantable ICD stands as the primary therapeutic choice [97]. Individuals who successfully overcame a prior cardiac arrest and/or those with documented instances of spontaneous sustained VTs, with or without syncope, were recommended as suitable candidates for receiving implantable ICDs as a secondary preventive measure against cardiac arrest [6]. Despite being an invasive procedure associated with frequent complications, such as inappropriate shocks because of T wave oversensing, infection, or psychological consequences, it remains the preferred course of action [97,98]. 

Pharmacological therapy can be implemented as an alternative to ICD in young patients or patients with contraindications, as well as additional support to prevent AF. Hydroquinidine (HQ) extends the QT interval in SQTS patients, though its impact on reducing cardiac events remains uncertain. HQ acts as a multichannel blocker, influencing the I_Kr_ channel to prolong the action potential duration and subsequently extend the QTc interval. Interestingly, despite sotalol and amiodarone being recognized as typical QT-prolonging drugs that also block I_Kr_, they proved ineffective in treating patients with SQTS1 [99,100]. Research indicates that the N588K site mutation in the HERG channel reduces channel inactivation, leading to an elevation in whole-cell channel current at physiological voltages by shifting the voltage dependence of inactivation to the right [101]. In a separate study, it was observed that introducing double mutations in the HERG channel amplifies the disruption of both channel gating and the channel’s response to drugs [D]. Specifically, when N588K and S631A mutations coexisted, the attenuation of channel inactivation and the impact of HQ were significantly more pronounced compared to instances where only N588K or S631A mutations were present individually. In summary, these findings collectively suggest that mutations in an ion channel can influence both channel gating and the effects of drugs.

The influence of HQ on asymptomatic patients was studied by Giusetto et al. [102]. The study included 53 participants and demonstrated efficacy in preventing the initiation of VA during both treatment and long-term follow-up. Additionally, 23% of participants expressed genetic mutations *KCNH2, HERG,* and *CACNB2b*. Patients with *HERG* mutation showed a better response to HQ treatment, presenting with normalization of the QT and effective refractory period; individuals lacking the mutation exhibited a less pronounced effect, with notable variability in responses [102]. HQ was reported to effectively lower the risk of SCD in SQTS. However, its administration may be associated with side effects, including gastrointestinal intolerance, leading to the discontinuation of treatment in up to 12% of cases [103]. To date, the literature describes 55 cases of SQTS patients treated with HQ in total. Only 41 individuals reported continuous usage during a median follow-up period of 5.6 years. Before the initiation of HQ, thirteen patients experienced one or more VA events. Following HQ treatment, VAs decreased significantly (*p* < 0.001). There were no noteworthy changes in the rates of AF. Fourteen patients discontinued the treatment, citing reasons such as gastrointestinal intolerance (*n* = 4), poor compliance (*n* = 8), and the absence of QTc prolongation (*n* = 2) [99].

Other antiarrhythmic medications such as disopyramide, nifekalant, quinidine, flecainide, sotalol, and ibutilide were also tested on a small cohort of SQTS1 patients without any apparent embitterment [104,105]. Nevertheless, a QTc prolongation in ECG was observed with the combination of digoxin and dofetilide, as well as with amiodarone and metoprolol. Additionally, disopyramide at the higher dosage (400 mg/day) also resulted in QTc prolongation in ECG. What is more, digoxin plus dofetilide and amiodarone plus metoprolol showed efficacy in preventing arrhythmias in SQTS patients [99].

Recently, experimental therapies such as optogenetic modulation have been proposed. Optogenetics enables the expression of light-sensitive proteins that act as ion channels. In the context of cardiology, it allows to modulate the cardiac muscle function by means of AP. Its utility in SQTS was examined on both cellular and tissue levels; it showed effectiveness in preventing reentry in SQTS or generally suppressing cardiac electrical activity. Although there is currently no translation of the method to an in vivo heart model, the study by Gruber et al. provides a promising outlook for future developments [106].

### 3.3. Brugada Syndrome (BrS)

#### 3.3.1. Epidemiology

BrS, named after Josep and Pedro Brugada, who initially identified it in 1992, is a hereditary condition marked by abnormalities in cardiac conduction. These irregularities were revealed as a right bundle branch block and ST-segment elevations in the right precordial leads (V1–V3) on an ECG stem from genetic alterations in transmembrane ion channels responsible for generating APs [107]. This genetic condition heightens the risk of life-threatening cardiac arrhythmias. Clinical manifestations vary, with sudden death typically occurring around the age of 40, though the syndrome can present from infancy to late adulthood. Moreover, it may be associated with sudden infant death syndrome (SIDS) and sudden unexpected nocturnal death syndrome (SUNDS), particularly in individuals from Southeast Asia. BrS is also linked to other conduction abnormalities such as first-degree AV block, right bundle branch block, intraventricular conduction delay, and sick sinus syndrome. Even though there are new theories about other ways of inheritance, BrS is still regarded as a condition inherited in an autosomal dominant manner, according to the most recent hypotheses. It is implicated in 4% of total SCDs and almost 20% of SCD cases in individuals with structurally normal hearts [108,109]. The global occurrence of BrS is estimated to be around 0.05%, while the more common Brugada pattern is observed in about 0.4% of individuals. In young adults without structural heart disease, BrS is linked to up to 20% of SCD, though this association is less significant in infants and children. BrS is considered to cause 10–20% of sudden infant deaths and 4–12% of SCD in children [110]. Males are more frequently impacted than females, constituting 80–90% of diagnosed cases, though this discrepancy becomes evident after adolescence. The highest prevalence of BrS is reported in Southeast Asia, where it is up to 14 times more common than the global prevalence. In this region, it is recognized as the primary cause of natural death among males below the age of 50. A small percentage of individuals affected by BrS undergo malignant arrhythmias, with a notably rare occurrence. The estimated annual incidence is 8–10% among patients with a history of VF, 0.5–2% in cases characterized by syncope, and 0–0.5% in asymptomatic groups. There is a pressing need for an effective risk stratification approach for the majority of patients who remain symptom-free throughout their lives. Consequently, it is imperative to unravel the complexities of the Brugada-type ECG pattern and its connection to the onset of tachyarrhythmia [111,112,113].

#### 3.3.2. Genetic and Molecular Aspects

The fundamental mechanism of BrS continues to be a matter of debate and contention. Two main hypotheses emerged: the depolarization disorder hypothesis and the repolarization disorder hypothesis. Both are shown in the Table 4 below [112,113]. Another less common theory, named neural crest theory, suggests that BrS may be associated with neural crest development abnormalities [114]. The right ventricular outflow tract, where electrical disturbances in BrS are claimed to occur, requires an extracardiac cell source from the neural crest during its development [114,115]. The overexpression, as well as underexpression, of connexin 43 (Cx43) is correlated with the faster and slower migration of cardiac neural crest, respectively. This may lead to heterogeneity of multiple connexins during the same time, subsequently causing the decreased conduction and late activation of RVOT observed in BrS [115,116]. Notably, these theories do not exclude each other and may have a synergistic role in BrS development [114].

**Figure 3:**
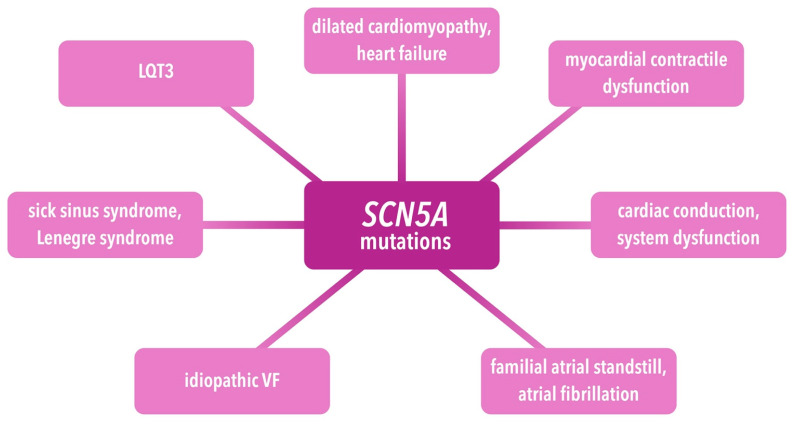
Cardiac conditions besides BrS are associated with SCN5A mutations [112,117].

BrS is inherited through an autosomal dominant transmission pattern. Mutations were discovered in 19 different genes, but only *SCN5A* is definitively linked to the disease, while the others are considered to have a less distinct association. These genetic mutations interfere with the normal operation of ion channels crucial to cardiac electrical activity, causing a reduction in inward sodium/calcium currents or an elevation in outward potassium currents. This disruption in the ionic balance during the initial phases of the AP contributes to the proarrhythmic nature of BrS [112]. Genes associated with BrS are listed in Table 5. *SCN5A* is situated on chromosome 3p21, and it encodes the subunit of the voltage-dependent Na^+^ channel (Nav1.5). The penetrance of *SCN5A* mutations was observed to exhibit incomplete penetrance and variable expression within pedigrees affected by BrS, indicating a complex inheritance pattern in which other genetic variants may impact the phenotype. Even individuals without the *SCN5A* genotype in affected pedigrees displayed the characteristic ECG pattern of Brugada type 1. *SCN5A* mutations extend beyond BrS and are associated with various other cardiac conditions. They are listed in the Figure 3 below [112,117]. 

#### 3.3.3. Diagnosis

**Figure 4:**
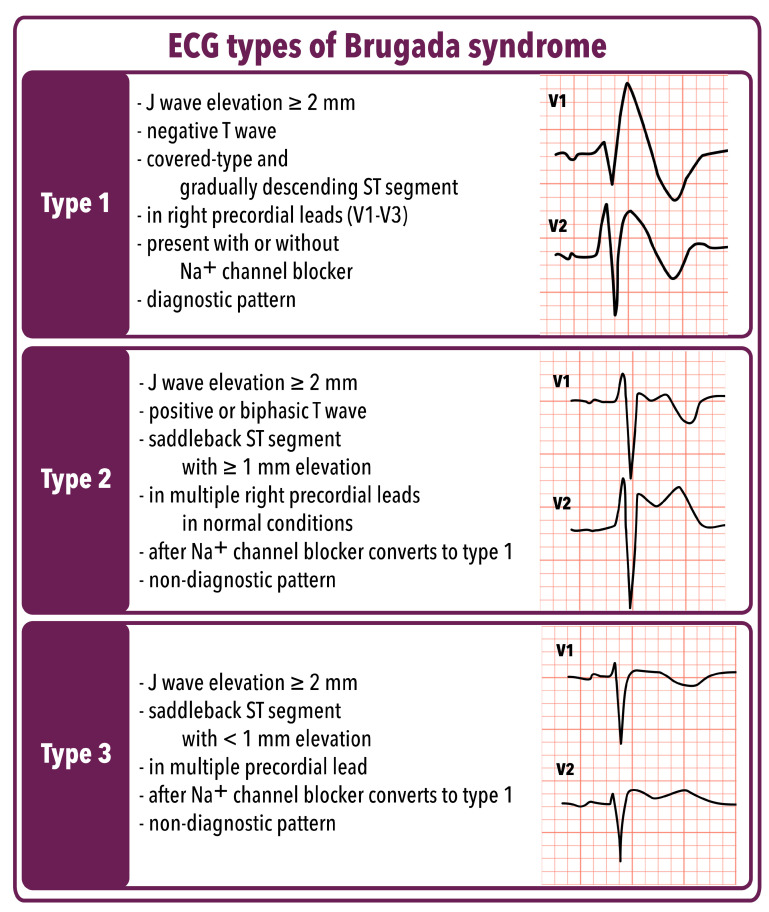
Electrocardiographic types of Brugada syndrome [122,123]. ECG—electrocardiography.

The clinical diagnosis of BrS is confirmed in an individual exhibiting distinctive ECG patterns along with indicative clinical history and/or family history. It should be considered in individuals presenting with any of the following indications: VF, recurrent syncope, cardiac arrest, self-terminating polymorphic VT, or a family history of SCD. Additionally, one of the types of ECG presented in Figure 4 should occur.

Ajmaline, an antiarrhythmic drug, is used as a pharmacological test for diagnosing BrS and identifying individuals with an elevated risk of experiencing life-threatening arrhythmias and SCD. Sodium channel blockers, including ajmaline, flecainide, or procainamide, can be administered to induce the type-1 BrS ECG pattern. Some practitioners prefer using ajmaline due to its higher sensitivity, which appears to result in a lower false-negative rate. Nevertheless, cautionary reports highlighted ajmaline’s potential for false positives, emphasizing that a positive ajmaline test does not always confirm the presence of BrS in a patient [118]. Numerous studies emphasized the intricate mechanism of ajmaline, indicating that it does not function solely as a sodium channel blocker. Instead, it also exerts additional effects on potassium and calcium channels [119]. Conducting an ajmaline test should be approached especially carefully because of the risk that a patient with a false-positive result could be mistakenly placed on BrS treatment [120].

According to the latest ESC Guidelines for the management of patients with patients with VA and prevention of SCD, BrS is identified in individuals without any concurrent heart conditions who exhibit a spontaneous type-1 Brugada ECG pattern. Also, the diagnosis of BrS is recommended in patients without any other heart disease who survived cardiac arrest caused by VF or polymorphic VT and display a type-1 Brugada ECG pattern induced by a sodium channel blocker challenge or during a fever. Genetic tests for *SCN5A* are recommended for probands with BrS [6]. A new scoring system, known as the Shanghai Score System, was developed for the diagnosis of BrS. Points are assigned for specific factors, including ECG changes, medical history, family history, and genetics. A score of 2–3 suggests a potential diagnosis of BrS, while a score of ≥3.5 confirms a definite diagnosis. The criteria and points assigned are shown in the Table 6 [122,123].

#### 3.3.4. Management

It is recommended for individuals with BrS to observe specific behavioral guidelines. These include refraining from the use of substances that could induce ST-segment elevation in the right precordial leads, such as cocaine and cannabis, as well as moderating excessive alcohol consumption [124]. 

It is advisable to refrain from using medications that potentially cause ST-segment elevation in the right precordial leads [6]. These drugs are divided into four groups: drugs to be avoided, drugs preferably avoided, potential antiarrhythmic drugs, and diagnostic drugs. The most relevant of these is the first group, which includes the following drugs: antiarrhythmic drugs (ajmaline, allpinin, ethacizin, flecainide, pilsicainide, procainmide, propafenone), psychotropic drugs (amitriptyline, clomipramine, desipramine, lithium, loxapine, nortriptyline, oxcarbazepine, trifluoperazine), anesthetics/analgesics (bupivacaine, procaine, propofol), and other substances (acetylcholine, alcohol, cannabis, cocaine, ergonovine) [121]. To verify which group a drug belongs to, the 2022 ESC Guidelines for the management of patients with VA and the prevention of sudden cardiac death recommend http://www.brugadadrugs.org (accessed on 20 December 2023) [6].

Moreover, it is essential to promptly address fever by using antipyretic medications [124]. In connection with the latter, Sivanandam et al. [122] described a noteworthy case of a patient hospitalized for intermittent fever associated with a non-productive cough. He did not have a background of fainting, irregular heartbeats, chest discomfort, difficulty breathing, or any other cardiovascular or pulmonary symptoms. There was no familial background of SCD or any other heart-related conditions. Laboratory results showed the patient had dengue fever. The patient was accompanied by recurrent fever during hospitalization. One of the tests performed was an ECG, which documented RBBB with ST-segment elevation. At discharge, the patient was stable and remained asymptomatic during follow-up. The ECG findings suggested BrS type-1, unmasked by dengue fever. This case illustrates that febrile illnesses, such us dengue fever, can reveal the presence of BrS in patients [122]. The results of studies proving a positive treatment effect in BrS patients are limited. The implantation of an implantable ICD is advised for individuals with BrS under the following conditions: those who have survived averted CA and/or those with documented instances of spontaneous sustained VT [6,125]. According to ESC guidelines, it is only in Class IIa that ICD implantation is suggested in patients with Brugada pattern type 1 and arrhythmic syncope [6,125]. Consideration should be given to the implantation of a loop recorder in BrS patients experiencing unexplained syncopal episodes. Transcatheter ablation should be regarded in patients with recurrent ICD discharges refractory to drug therapy. This is not recommended in asymptomatic patients with BrS syndrome. The use of quinidine should be considered in patients who are eligible for ICD implantation but have contraindications, decline the procedure, or experience recurrent ICD discharges. In patients with electrical storms, isoproterenol infusion needs to be considered [6,125].

### 3.4. Catecholaminergic Polymorphic Ventricular Tachycardia (CPVT)

#### 3.4.1. Epidemiology

CPVT is a genetic channelopathy disorder characterized by the presence of polymorphic VT and bidirectional VT caused by physical or emotional stress. Its epidemiology involves a low prevalence, estimated at around 1 in 10,000, with a mortality rate of up to 13% for patients under treatment [126]. CPVT was first described in 1978 [127]. It usually manifests in children and young adults, potentially leading to life-threatening arrhythmias. Approximately 30% of CPVT patients have a family history of SCD before the age of 40 [127]. There is no significant gender predilection, and both males and females can be affected. Understanding the epidemiology of CPVT is crucial for early identification, appropriate management, and genetic counseling for affected individuals and their families.

#### 3.4.2. Genetic and Molecular Basis

CPVT is typically caused by mutations in genes associated with calcium regulation in cardiac cells, such as the ryanodine receptor gene *(RyR2)* and calsequestrin gene *(CASQ2)*. Mutations in *RyR2* are characterized by an autosomal dominant pattern of inheritance; however, they can also be inherited recessively by mutations in the cardiac gene CASQ2 [128]. The *RyR2* gene mutation accounts for about 60–70% of all CPVT cases [129]. While the *CASQ2* mutation is less common (about 10–15%), it is more severe [129].

Normal heart function relies on tightly regulated calcium levels within cardiac cells. Calcium is essential for the contraction and relaxation of the heart muscle. In CPVT, mutations in specific genes disrupt the normal handling of calcium. Both genes are involved in the control of calcium release from the SER; *RyR2* is the SER calcium-releasing channel, and CASQ2 is a calcium-buffering protein that may also exert a regulatory function of *RyR2* [127]. Mutations in the *RyR2* and *CASQ2* genes lead to a leakage of Ca^2+^ from the SR in diastole, particularly under adrenergic stress, manifested by an increased risk of DAD and, therefore, vulnerability to abnormal heart rhythms in response to stress or exercise. These are leading to VA [130].

While *RYR2* and *CASQ2* are the primary genes associated with CPVT, there are additional identified genes that may contribute to the condition. Two potential genes currently being investigated are the calmodulin gene *(CALM1)* and the triadin gene *(TRDN)* [129]. *TRDN* gene is involved in the stabilization of calcium channels, and mutations can affect calcium regulation. Mutations in *CALM1*, which encodes a calcium-binding protein, can disrupt normal calcium signaling in cardiac cells. CPVT-associated *CALM1* mutations were shown to evoke arrhythmogenic Ca^2+^ waves in cardiomyocytes [126]. 

Regardless of the varying genetic mutations, CPVT cases present very similarly phenotypically [129]. However, de novo mutations can also contribute to CPVT. Genetic testing plays a crucial role in identifying these mutations, allowing for early diagnosis and appropriate management. It should be noted that not all individuals with CPVT will have an identifiable genetic mutation, and in some cases, the genetic basis remains unknown.

#### 3.4.3. Diagnosis 

The diagnosis of CPVT typically involves a combination of clinical, genetic testing, and cardiac imaging. Clinical features often occur during childhood and may include exercise-induced syncope, palpitations, or sudden cardiac arrest by adulthood [130]. All of them are mostly triggered by physical activity or emotional stress. Due to the wide spectrum of symptoms leading to many other tachyarrhythmias, CPVT diagnosis is, on average, delayed by two years from the first syncopal episode [129]. Genetic testing can identify mutations in genes associated with CPVT, such as the *RyR2* or *CASQ2* genes [128]. However, not all individuals with CPVT will have identifiable mutations. Holter monitoring and cardiac imaging like echocardiography may also be used to assess heart function and detect arrhythmias, but in the CPVT case, the presence of a structurally normal heart and ECG at rest is the most common [131]. The most effective form is exercise stress testing by a treadmill stress test [130]. Moreover, epinephrine or isoproterenol provocation test may be considered in patients unable to perform exercise tests [6]. An exercise test may show VA in the form of polymorphic or bidirectional VT [131]. The CPVT diagnosis is typically made in people aged under 40 years [131]. 

#### 3.4.4. Management

While potentially fatal, CPVT can be successfully managed if diagnosed early. The treatment of CPVT aims to prevent life-threatening arrhythmias and manage symptoms. One of the key aspects of CPVT treatment includes lifestyle modifications. Competitive and intensive leisure time sports are not recommended. Patients should also avoid stressful and emotional situations, dehydration, electrolyte disturbance, and hyperthermia [131]. 

Another indispensable form is pharmacological treatment. The life-long administration of β-blockers is currently the first-line therapeutic option for CPVT patients [129]. β-blockers help reduce the response to catecholamines, which can trigger arrhythmias in individuals with CPVT. Recent studies demonstrated that nadolol is the most effective beta blocker in CPVT [131]. It is used in a high dosage of 1–2 mg/kg [130]. Carvedilol may be a good choice because of its recently discovered direct *RyR2*-blocking properties, although clinical data are lacking [127]. Moreover, even asymptomatic gene carriers should be treated at least with a β-blocker [128]. 

Prognostication in CPVT involves assessing the likely course and outcome of the condition for each individual. In contrast to other inherited arrhythmia syndromes, such as LQTS and BrS, there are fewer risk factors that provide prognostic information in patients with CPVT [127]. If untreated, CPVT is highly lethal, as approximately 30% of affected individuals experience at least one cardiac arrest, and up to 80% have one or more syncopal spells. Sudden death may be the first manifestation of the disease [132]. As the understanding of CPVT continues to evolve, individualized care plans based on a comprehensive assessment of genetic, clinical, and lifestyle factors will contribute to more accurate prognostication in the future.

### 3.5. Idiopathic Ventricular Fibrillation (IVF)

#### 3.5.1. Epidemiology

SCD usually occurs within an hour of the onset of symptoms and is caused by heart disease [133]. In developed countries, it is the leading cause of death [134,135]. 

Patients who survive SCD by VF and have an apparently normal heart structure were classified as IVF [61,136]. IVF in young patients without electrical or structural heart disease is considered a major cause of SCD. IVF is caused by Purkinje fiber-derived short-coupled premature ventricular complexes descending on the T-wave, called the R-on-T phenomenon [137]. Over the past 30 years, the definition of IVF has changed through the discovery of genetic heart disease or the introduction of molecular autopsy, making IVF an exclusionary diagnosis. IVF is diagnosed in patients, after sudden cardiac arrest (SCA), who have been rescued and in whom a VF rhythm was documented while excluding metabolic, respiratory, cardiac, and toxicologic etiologies. The population of patients who survived SCA, unexplained by any means, varies depending on the study [61].

Less than 7% of all patients are those who survived out-of-hospital cardiac arrest and received a diagnosis of IVF [134]. In the Asian cohort of patients with implantable cardioverter–defibrillator after SCA with normal ECG, the incidence is just over 8% and almost 11% in Caucasians [61].

Despite the fact that IVF is an exclusionary diagnosis, we can find several elements in common among the patient group. First of all, they are young men, and the primary arrhythmia, if documented in any way, is VF-triggered by short-coupled premature ventricular complexes (PVCs) or polymorphic VT [138]. Regarding gender differences, and thus, the gender differences in arrhythmia risk predisposed to SCD, female sex hormones were found to be arrhythmogenic [139]. The work raises, therefore, the question of whether sex hormones are responsible for the automaticity of Purkinje fibers. In men, Purkinje ectopy originated in the vast majority from the right ventricle, while in women, it originated from the left or both. Conterminous phenomenon has been observed in AF, where the arrythmia originated from pulmonary veins mostly in men, whereas in woman the origin was predominantly localized extrapulmonary veins. The influence of sex hormones seems important. They regulate the transcription, expression, post-translational modification, and function of Ca21 protein, contributing to differences in cardiac arrhythmogenesis. Work in animal models suggests that female sex and the presence of estradiol cause an increase in L-type calcium current. The topic of estradiol’s effect on the regulation of Ca21 handling proteins and the expression of ion channels and Ca21 in Purkinje fibers requires further study [139].

#### 3.5.2. Genetic and Molecular Basis

According to the latest European Heart Rhythm Association (EHRA)/Heart Rhythm Society (HRS), American Heart Association (AHA), and American College of Cardiology (ACC) guidelines, genetic testing is recommended for patients who survived SCA from unexplained causes, and therefore, the default diagnosis is IVF if there is a clinical suspicion of genetic heart disease (GHD) [61]. In general, the use of genetic testing in large centers for patients who survived SCA is low and results in giving a default diagnosis of IVF. Within the period of 2017 to 2018, a study was published in which the authors sequenced exomes. The occurrence of one or more variants of unknown significance (VUS) in channelopathy surpasses the incidence of pathogenic/likely pathogenic (P/LP) variants. In addition, the lack of the patient’s clinical phenotype and poor accessibility to the patient’s family history create the problem of misdiagnosis. It is, therefore, worth mentioning that genetic testing should be carried out in highly specialized cardiovascular genomics centers to avoid interpretation errors. The following genes responsible for IVF were discovered, as shown in the figure below [61].

**Figure 5:**
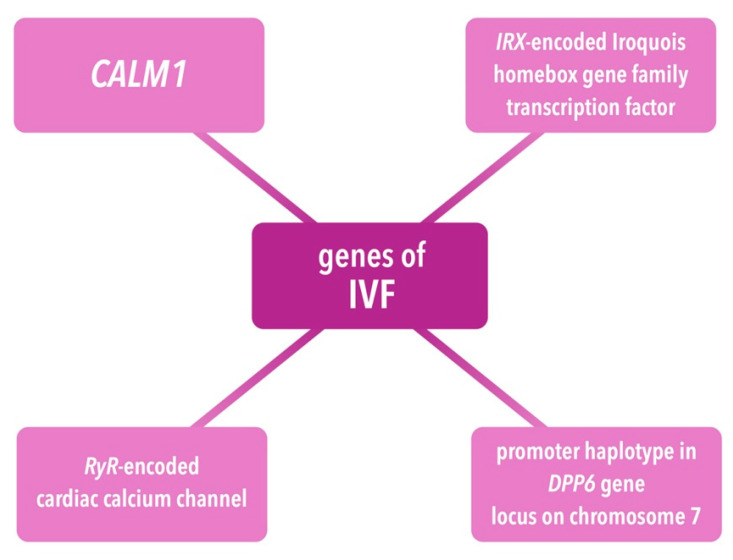
Genes of idiopathic VF [61].

The *DPP6* haplotype plays a diagnostic role in the fact that, in the study, it showed more than 20 times higher levels in IVF patients than in the group of healthy patients studied. In addition, in children, LQT/CPVT mutations, in which calmodulin/tridine is indirectly involved, were checked in connection with exercise-induced cardiac arrest. In view of these facts, there will be a need for more specialized tests for patients, which will also screen family members. On the other hand, it is worth mentioning that there is still a lack of specific guidelines for evaluating families of patients after unexplained cardiac arrests [61]. Genes of idiopathic VF are presented in Figure 5.

#### 3.5.3. Diagnosis

In view of the exclusionary diagnosis of IVF, a number of tests should be performed on the patient. Starting with a 12-lead ECG, which will allow us to rule out cardiac abnormalities such as QT syndromes or early J-wave repolarization syndromes [140]. The next test is Holter cardiac monitoring [140,141]. We can perform provocative tests for latent mutations in the sodium or potassium channel or an exercise treadmill test. Additional imaging tests that we can perform include two-dimensional echo, coronary angiogram, or cardiac MRI [140]. 

Tests that involve drug administration are the intravenous epinephrine test and the procainamide challenge test. Patients also undergo invasive tests such as electrophysiological testing or myocardial biopsy [61]. It should be noted that current guidelines recommend diagnosis in patients with unexplained cardiac arrest (UCA) to detect the cause, but diagnosing IVF does not have strict requirements for diagnostic tests. This raises a lot of controversy because the diagnosis is inaccurate, and the definition of the disease itself by exclusion is imprecise. The highest-performing tests that should be an absolute minimum are cardiac magnetic resonance imaging (CMR), exercise treadmill test (ETT), and sodium channel blocker (SCB) [141]. Moreover, genetic variants associated with cardiomyopathy may be involved in causing IVF, so they should be looked at in the future as a likely risk factor [142].

#### 3.5.4. Management

According to the ESC 2022 guidelines, we have several treatment options for IVF patients [6]. We can implant an implantable ICD in the case of IVF, and this is a class I level B recommendation. Pharmacotherapy, which is an infusion of isoproterenol, verapamil, or quinidine jet for the acute treatment of electrical storm or recurrent ICD discharges, is a Class IIa level C recommendation. We can also consider the chronic use of quinidine to suppress electrical storms or recurrent ICD discharges in IVF, and this constitutes a class IIa level B recommendation. The last possible method is percutaneous catheter ablation by electrophysiologists with extensive experience in patients with recurrent IVF with repeated episodes of VF, called unresponsive to pharmacotherapy, with similar PVCs constituting class IIa and level C [6]. Therefore, it is important to correctly diagnose IVF in order to undertake selected treatment and enable patients to maintain a life of satisfactory quality and level.

### 3.6. Early Repolarization Syndrome (ERS)

#### 3.6.1. Epidemiology

The 2022 ESC Guidelines for the management of patients with VA and the prevention of sudden cardiac death described early repolarization syndrome (ERS) as the occurrence of an early repolarization pattern (ERP) in individuals who were successfully resuscitated from a confirmed instance of unexplained VF or polymorphic VT [6].

While historically considered a minor phenomenon, more recent studies linked ERP features to a greater risk of life-threatening arrhythmias and SCD, which is associated with specifying ERS as a medical condition, consequently contextualizing the finding of ERP within the need for increased vigilance due to its potential clinical significance [143,144].

The prevalence of ERP in the general population displays variability based on different studies and specific populations, spanning a range from 1.3% to 13.3%, although meta-analytical studies reported prevalence figures for the general population between 6.7% and 11.6% [145,146,147,148,149,150,151]. Prevalence variations exist among different racial groups [145,149,152]. Notably, ERP exhibits a higher prevalence in physically active individuals, reported as reaching 33.9% [145]. Research on ERS consistently indicated a higher prevalence among men [153,154,155,156,157] and a younger age at diagnosis [154,156]. Identified independent risk factors associated with ERP include younger age, male gender, lower systolic blood pressure (SBP), shorter QRS duration, a shorter QTc interval, and hypothermia [145,146,151]. 

Observational studies demonstrate varying associations of ERP with different cardiac conditions. Some studies observed a higher prevalence of ERP in specific conditions, such as left ventricular hypertrophy, or among survivors of SCD with idiopathic VF [158,159,160]. Another study revealed 2.1% cardiac mortality during a mean follow-up of 14.2 ± 2 years after the detection of ERP [147]. A subsequent meta-analysis reported an increased risk of SCA (RR 2.18; 95% CI 1.29–3.68), cardiac death (RR 1.48; 95% CI 1.06–2.07), and all-cause death (RR 1.21; 95% CI 1.02–1.42) among individuals with ERP, estimating that it was responsible for 7.3% of SCA in the general population [148]. Furthermore, ERP was associated with a heightened risk of arrhythmia-related mortality (HR 1.70; 95% CI 1.19–2.42; *p* = 0.003), although in this study there was an insignificant impact on cardiac mortality (HR 0.78; 95% CI 0.27–2.21; *p* = 0.63) or all-cause mortality (HR 1.06; 95% CI 0.85–1.31; *p* = 0.62) [149]. Moreover, an age-stratified analysis indicated hazard ratios of 1.96 for cardiac mortality (95% CI 1.05–3.68; *p* = 0.035) for both sexes and 2.65 (95% CI 1.21–5.83; *p* = 0.015) for men aged 35–54 years. Furthermore, an inferior localization of ERP augmented ERP-associated cardiac mortality (HR 3.15; 95% CI 1.58–6.28; *p* = 0.001 for both sexes and HR 4.27; 95% CI 1.90–9.61; *p* < 0.001 for men aged 35–54 years) [150]. The presence of ERP in these settings might indicate underlying cardiac structural abnormalities or susceptibility to certain arrhythmias, although the exact nature of this relationship requires further investigation.

#### 3.6.2. Genetic and Molecular Basis

ERS remains a complex and incompletely understood medical entity. While recommendations advocate its diagnosis through electrocardiographic findings and exclusionary criteria [6], addressing ERS continues to pose diagnostic and therapeutic challenges for clinicians. Extensive studies have been conducted to comprehensively investigate the disease and enhance the understanding of its essence. 

Two mechanistic theories concerning ERS were proposed, encompassing molecular studies and investigations on animal models [161]. Primarily, ERS was largely considered a repolarization anomaly. The initial hypothesis presented by Osborn speculates on the appearance of the J-wave, suggesting a “current of injury” [162]. Numerous studies subsequently detailed the epicardial phase 1 AP notch and transmural heterogeneity, correlating these findings with arrhythmogenesis (to be discussed in the subsequent sections). At the same time, alternative theory suggests depolarization abnormalities, drawing parallels to the clinical similarities observed in BrS. This hypothesis, in part, involves disturbances in currents and altered conduction while also considering structural abnormalities [161]. Nevertheless, there are reports that local depolarization heterogeneity may have a minor contribution to arrhythmogenesis [163]. 

Moreover, Miles et al. [164] presented a hypothesis suggesting that ERS could be potentially placed within the spectrum of subepicardial cardiomyopathy. Their theory revolves around the convergence of genetic predisposition, ionic imbalances, and environmental vulnerabilities that collectively impact the subepicardial conduction reserve. This depletion creates a mismatch between electrical current and load in areas with minor microstructural changes, culminating in the formation of a substrate for arrhythmogenesis that can lead to the manifestations of ERS [164]. On structural abnormalities standing for ERS, bearing comparison to BrS, Boukens et al. have also made speculations [165].

Overall, ERS presents an intricate interplay of molecular mechanisms involving ion channel mutations and alterations, influencing cardiac repolarization, particularly changes in potassium current and fluctuations in natrium and calcium currents levels, which is about to be discussed in this chapter.

##### Potassium Currents

A study conducted on rabbit hearts showed that the activation of a small-conductance calcium-activated potassium (SK) channel resulted in a proarrhythmic effect. SK3 channels were identified as the dominant subtype involved in the development of J-wave syndromes (JWS) that are dependent on the SK current (I_SK_). The simultaneous activation of I_SK_, while inhibiting the sodium current, resulted in enhanced AP dynamic instabilities. This included a shorter AP duration, repolarization alternans, slowed intraventricular conduction velocity, and spontaneous VF [166,167]. Mapping studies showed that the initiation of phase 2 reentry occurred predominantly at epicardial sites [166]. 

Other research indicated the involvement of the colocalization of SK channels with L-type calcium channels in the elevation of J-waves and reentry mechanism. The proximity of these channels results in the higher exposure of SK channels to intracellular Ca^2+^ levels, modifying potassium currents. Computer simulation [168] suggested that, in this configuration, I_SK_ may track an intracellular Ca^2+^ transient, synergistically interacting with I_to_. A spiky Ca^2+^ transient in subsarcolemmal space and junctional clefts was found to stimulate an I_SK_ spike-like pattern during APs. This phenomenon correlated with AP duration alternans, phase 2 reentry, J-wave elevation, and potential arrhythmias, consistent with other studies on rabbit heart models [167,168]. 

Additionally, Fei et al. [167] highlighted the stimulating effect of acetylcholine on ventricular I_SK_, particularly in men. This finding aimed to explain the occurrence of SCD during periods of high parasympathetic activity, especially at night [167]. The suppression of I_SK_ demonstrated antiarrhythmic effects similar to β-adrenergic activity induced by isoproterenol. Conversely, hypothermia, which was shown to stimulate I_SK_, had adverse proarrhythmic effects [166]. In contrast, Chen et al. [168] reported significantly enhanced I_SK_ after β-adrenergic stimulation in female rabbit ventricles. This led to negative intracellular Ca^2+^–voltage coupling and facilitated VF [169]. 

Transient outward potassium current (I_to_) is responsible for early repolarization. An alteration in I_to_ is indicative of changes in cellular repolarization, potentially contributing to the arrhythmogenic substrate observed in ERS. A heterozygous de novo mutation in the *KCND3* gene, encoding potassium voltage-gated channel subfamily D member 3 (K_v_4.3), was identified in an 8-year-old boy with ERS, attributed to gain-of-function changes in K_v_4.3. The mutation led to altered inactivation and recovery from inactivation, along with a 1.6-fold increase in I_to_ [170]. The *KCND3*-V392I variant, due to enhanced expression, increased I_to_ density by 60.9% [171]. This mutation was also found in siblings presenting cardiocerebral phenotypes, including ERS [172]. Another case concerned the involvement of *KCND3* duplication [173]. Additionally, Teumer et al. [174] proved 43 genome-wide significant Single-Nucleotide Polymorphisms (SNPs) at the *KCND3* locus to be highly associated with ERP. 

A genomic analysis of four unrelated Chinese families with ERS revealed a *DPP6*-L747P variant of the dipeptidyl aminopeptidase-like protein-6 (DPP6), which is an accessory subunit of I_to_ channels. This variant possesses the capacity to bind to and modulate the expression of voltage-gated potassium channels. Subsequent experimental studies on HEK 293 cells showed that the *DPP6*-L747P variant led to a gain-of-function in the I_to_ current, which was functionally expressed as enhanced current density [175]. *DPP6* was previously linked to idiopathic VF and is suggested to pose an increased risk of both VF and SCD [176,177]. 

Furthermore, mutations affecting the subunits that constitute the ATP-sensitive potassium (K_ATP_) channel, leading to gain-of-function modifications, are considered contributors to arrhythmogenic susceptibility. The *KCNJ8* gene, responsible for encoding the Kir6.1 subunit, is assumed to be implicated in the molecular progression of ERS. The S422L-*KCNJ8* mutation found in a patient with ERS demonstrated a gain-of-function effect on K_ATP_ current (I_K-ATP_) [178,179]. It was noted that this mutation led to decreased sensitivity of the channel to intracellular ATP, contra-acting the inhibition of channel opening and, thereby, enhancing I_K-ATP_ [178]. Similarly, a gain-of-function in I_K-ATP_ was observed in a highly conserved mutation in *ABCC9*, which encodes SUR2A, a regulatory subunit of K_ATP_. This specific mutation was thought to alter the sensitivity of I_K-ATP_ to ATP, thereby affecting the channel’s behavior and contributing to the observed changes in potassium current [180].

What is more, a gain-of-function missense mutation in the *hERG/KCNH2* gene, encoding an α-subunit of the rapid component of the delayed rectifier K^+^ (I_KS_) channel, which contributes to later repolarization, was detected in a Chinese family with ERS-linked SCD. Further analysis showed that the mutation enhanced the peak tail potassium current and accelerated activation and deactivation rates [181]. I_KS_ current could also be affected through the decreased expression and membrane trafficking of the β-subunit of the K_v_ channel-KCNE1 protein [182]. 

##### Sodium Currents

Pathogenic variants in *SCN5A*, which encodes the sodium channel Na_v_1.5, were found in a subset of ERS patients, consequently resulting in reduced sodium current (I_Na_), a crucial component in AP generation and propagation. Notably, in a study conducted by Zhang et al., pathogenic variants of *SCN5A* were discovered in 10 out of 104 patients diagnosed with ERS [154]. Specifically, the *SCN5A* mutation C280S*fs61 resulted in the loss of sodium channel functionality due to the production of a severely truncated protein. In the heterozygous state, this mutation led to a halved current density, indicating a haploinsufficiency effect [183]. Additionally, the G4297C mutation was found to inhibit the expression of sodium channels in the membrane, consequently reducing I_Na_. Interestingly, the co-occurrence of a specific polymorphism in the same allele appeared to mitigate the deteriorating effects of the G4297C variant [184]. Another loss-of-function mutation, the A1055G variant, demonstrated significantly decreased I_Na_, which might be correlated with a relatively increased outward current during early repolarization, potentially contributing to arrhythmogenesis [185]. Further, the L1412F mutation, identified in a patient with a first-time reported fever-induced ERS, resulted in the destabilization of the Na_v_1.5 structure. Similarly, the G452C variant, only when co-expressed with wild-type KCND3, notably altered the peak I_Na_ by 44.52% at 20mV, simultaneously influencing I_to_ with observed 106.81% rise in I_to_ density at +40mV [154]. 

Also, the glycerol-3-phosphate dehydrogenase 1-like (*GPD1-L*) gene was implicated in ERS pathogenesis, acting via regulation of the function of cardiac sodium channels. The *GPD1-L* P112L mutation correlated with decreased membrane distribution of GPD1-L protein and was associated with reduced I_Na_ activation [186]. Another study described a 60% reduction in I_Na_, associating the loss of current density with the plakophilin 2 (*PKP2*) gene mutation, specifically the *PKP2* D26N mutation, previously linked to BrS. This protein is known to influence Na_v_1.5 by its association with ankyrin G [187]. This observation highlights its potential pathogenic role in ERS.

Compound interactions in ion currents were described. Mutant variants in the sodium channel beta-1b subunit (*SCN1Bβ*), namely S248R and R250T, detected in ERS patients, were found to increase I_to_ when co-expressed with *KCND3*, respectively, by 27.44% and 199.89%, compared to wild-type. Physiologically, the mutations affected the gating kinetics of K_v_4.3 with a diminished steady-state inactivation and recovery from inactivation. However, they did not affect I_Na_ when co-expressed with wild-type *SCN5A*. Further analysis revealed a greater association between both *SCN1Bβ*-mutated variants and K_v_4.3, which could explain the modulatory effect of *SCN1Bβ* on the gating kinetics of K_v_4.3 with consequent I_to_ stimulation [188]. 

##### Calcium Currents

A significant discovery in the understanding of ERS pathogenesis involves loss-of-function mutations identified in the α1, β2, and α2δ subunits of the cardiac L-type calcium channel (LTCC), denoted as *CACNA1C, CACNB2,* and *CACNA2D1* genes, respectively.

Burashnikov et al. [156] were the first to report calcium channel mutations to be involved in ERS pathogenesis. In a cohort of 24 individuals with ERS, four probands were found to possess mutations in calcium channel genes, while three individuals exhibited rare polymorphism affecting LTCC [156].

Another genomic analysis of 104 ERS probands further revealed defects in calcium channel genes in 16 unrelated patients (32.2 ± 14.6 years old, 87.5% male). Notably, half of these patients carried more than one susceptibility gene variant for ERS. Loss-of-function mutations in the cardiac calcium channel genes in those cases were associated with decreased heart rate, shorter QTc interval, and increased transmural dispersion of repolarization. Laboratory studies showed that mutations, such as *CACNA1C*-P817S, resulted in impaired membrane targeting of the Ca_V_1.2 protein, the primary subunit of the LTCC, leading to a reduction of 84.61% in the density of the calcium current (I_Ca_). The heterogeneous expression of affected channels reduced I_Ca_ by 51.35%. This decrease in I_Ca_ intensified net outward currents and accelerated cardiomyocyte repolarization [189]. Moreover, an investigation into a Chinese family experiencing SCD associated with ERS revealed a hereditary loss-of-function Q1916R mutation in the *CACNA1C* gene, which showed a 60% penetrance rate within the affected family members. Interestingly, the phenotypic expression of calcium channel mutation carriers could be modulated by the presence of the gain-of-function *SCN5A*-R1193Q variant. This variant led to a persistent inward tail I_Na_, potentially exerting a protective effect. The interplay between the loss-of-function calcium channel mutation and the SCN5A variant, along with other genetic factors and sex, suggested a complex oligogenic background influencing disease manifestations in ERS [155].

These studies collectively emphasize the intricate role of calcium channels and their influence on cardiac electrophysiology, potentially contributing to arrhythmogenesis.

The pathogenesis of ERS appears to involve a compound genetic background. There are reports of more severe ERS phenotypes in a case where there were gain-of-function mutations in I_K-ATP_, and late I_Na_ currents coexisted with a loss-of-function mutation in peak I_Na_. [180]. 

Moreover, carriers of multiple mutations in cardiac arrhythmia-associated genes may manifest overlapping syndromes. For instance, a patient with six pathogenic mutations—*RYR 2, KCNA5, PKP2, ANK2, HCN2,* and *ABCC9*—showed a combined phenotype of SQTS and ERS. While *ABCC9* was previously linked to ERS, a mutation in *PKP2* was suggested as the second mutation contributing to ERS phenotype [187]. In another case, the co-occurrence of three mutations, *SCN9A, FKBP1b,* and *PXDNL*, was detected in a patient exhibiting features of both ERS and BrS [190].

In summary, molecular findings in ERS predominantly revolve around mutations affecting potassium, calcium, and sodium channels (Table 7), resulting in changes in current densities, channel structure, and function. These alterations contribute to the disruption of normal cardiac electrical activity, thereby predisposing individuals to arrhythmic events seen in ERS. Understanding these molecular mechanisms is crucial for developing targeted therapeutic interventions and improving risk stratification in individuals affected by ERS. Indeed, while there’s a growing body of evidence derived from experimental models, particularly animal models and cellular cultures, our understanding of the primarily human cardiac environment remains limited. This suggests that there is still much to uncover and many aspects yet to be revealed about this complex entity.

#### 3.6.3. Diagnosis

Life-threatening heart arrhythmias frequently occur unexpectedly as the initial presentation of ERS. It is important to note that this condition is often asymptomatic and diagnosed incidentally through ECG findings or after a cardiac event. Some patients present with a history of syncope or have a family history of SCD at a young age, idiopathic VF, or ERP [156]. 

The diagnosis of ERS mandates specific criteria to be met [6]:

(1) A patient resuscitated from otherwise unexplained idiopathic VF or polymorphic VF or an SCD victim with a negative autopsy report;
(2) Documented ECG displaying ERP: J-point elevation ≥1mm in ≥2 adjacent inferior and/or lateral leads.

Other criteria propose considering the clinical evaluation of first-degree relatives of ERS patients and conducting genetic testing in ERS patients [6]. 

Early repolarization pattern is a common finding in ECGs and is typically considered a normal variant. It is characterized by J-point elevation, often seen as terminal QRS slurring or notching, coupled with ST-segment elevation displaying an upper concavity, along with prominent T-waves in at least two contiguous leads [143]. However, there is ongoing debate and modifications regarding its definition due to inconsistencies in describing ST segment and J-waves, leading to varying interpretations and controversies over the appropriateness of the use of the term “early repolarization” [144,191,192]. Some undermine the use of the term “ST-elevation”, highlighting that it might not adequately capture the diversity of ST segment presentations, proposing a focus on detailed J-point changes or expanding the definition to specify early repolarization with ST-segment elevation or terminal slur/notch [144]. Research indicates that J-point elevation ≥ 0.1 mV in the inferior leads, coupled with notching configuration, significantly increases the risk of arrhythmia-related death [149]. Another research work points out the need for the proper differentiation of J-waves with pseudo-J-waves entailed by terminal QRS deflection in depolarization abnormalities [193]. 

The Shanghai Score System was proposed as a diagnostic tool for ERS. It evaluates the clinical history, ECG findings, family history, and presence of ERS susceptibility mutations [194]. It has already been reported to effectively identify ERS patients prone to recurrent VF [195].

Differential management is crucial, especially in differentiating BrS from ERS, as both fall under J-wave syndromes due to their similar characteristics. However, they are suspected to have distinct pathomechanisms [153]. In differentiating ERS from BrS, the application of pilsicainide or ajmaline, used as a pharmacological sodium-channel blocker challenging test, yields negative results in ERS patients [153,196]. Additionally, Kamakura et al. [197] proposed a spasm provocation test to distinguish ERS from silent coronary artery spasm.

#### 3.6.4. Management

2022 ESC Guidelines for the management of patients with VA and the prevention of sudden cardiac death [6] compiles the following recommendations for the management of patients with diagnosed ERP/ERS:

- Class I recommendations: ICD implantation is recommended in patients with a diagnosis of ERS who survived cardiac arrest;
- Class IIa: isoproterenol infusion should be considered for ERS patients with an electrical storm; quinidine in addition to an ICD should be considered for recurrent VF in ERS patients; implantable loop recorder should be considered in individuals with ERP and at least one risk feature of arrhythmic syncope; premature ventricular complexes (PVC) ablation should be considered in ERS patients with recurrent VF episodes triggered by a similar PVC non-responsive to medical treatment;
- Class IIb: ICD implantation or quinidine may be considered in individuals with ERP and arrhythmic syncope and additional risk features; ICD implantation or quinidine may be considered in asymptomatic individuals who demonstrate a high-risk ERP in the presence of a family history of unexplained juvenile sudden death;
- Class III: ICD implantation is not recommended in asymptomatic patients with an isolated ERP.

It should be noted that the 2015 ESC Guidelines for the management of patients with VA and the prevention of sudden cardiac death refrained from issuing recommendations regarding ERS management, justifying this by insufficient evidence [198]. 

On the other hand, the 2017 AHA/ACC/HRS Guideline for the management of patients with VA and the prevention of sudden cardiac death [199] recommends the following:

- Class I recommendations: Observation without treatment in asymptomatic patients with ERP; ICD implantation in patients with ERP and cardiac arrest or sustained VA if meaningful survival >1 year is expected;
- Class III: Genetic testing in patients with ERP is not recommended.

Regarding the pharmacological approach, PDE3 inhibitors, such as cilostazol and milrinone, known for their action on cardiac calcium channels, show promise in addressing repolarization abnormalities. These agents demonstrate the ability to restore the AP dome specifically at epicardial sites, thereby reducing both the epicardial and transmural dispersion of repolarization. In canine left ventricular myocytes, cilostazol reduced I_to_ by 44.4%, while milrinone decreased it by 40.4% at +40 mV [200,201]. 

Isoproterenol, a non-selective β-adrenoceptor agonist, was proven effective in mitigating hypothermia-induced electrical storms in ERS patients and suppressing arrhythmic activity [155,166,200,202]. 

Moreover, the application of quinidine showed inhibitory effects on potassium and sodium currents, acting preventatively against VF recurrence and reducing the number of ICD shocks in patients with implanted ICDs [170,201,203,204]. Serum quinidine levels were observed to correlate with J-wave amplitude on ECG [205]. However, some clinicians suggest restricting quinidine use to short-term therapy in patients with limited therapeutic options due to frequent gastrointestinal intolerance [204]. Additionally, there are isolated reports on the effective suppression of electrical storms using orciprenaline, acting as an I_to_ inhibitor [206].

Ongoing scientific endeavors involve the evaluation of novel therapeutical approaches. Acacetin, a natural flavone, shows promise in reversing repolarization abnormalities via inhibiting I_to_, thereby suppressing VF [207]. It was shown to effectively block peak I_to_ current density in associated gain-of-function *KCND3* mutation [171]. ARumenamide-787 was proposed as a potential agent targeting arrhythmogenesis in JWS patients. Experimental studies utilizing canine ventricular wedge preparations demonstrated its efficacy in restoring homogeneity of repolarization by suppressing I_to_ and stimulating I_Na_, with limited adverse effects [208]. 

Catheter ablation represents an important therapeutic option for individuals with recurrent VF despite not achieving full efficacy due to targeting challenges [206,209,210]. In an experimental model, the abnormal repolarization of the epicardial AP in the susceptible area in the myocardium was detected with bipolar electrograms showing low-voltage fractionated potentials. These were associated with phase 2 reentry and high-frequency late potentials, potentially suitable for radiofrequency ablation [211]. High-density electrophysiologic mapping holds promise in identifying substrates of arrhythmias [209,210,212].

## 4. Conclusions

Primary electrical heart diseases, comprising channelopathies, are inherited genetic abnormalities of cardiomyocyte electrical function with the absence of structural abnormalities. LQTS, SQTS, BrS, IVF, CPVT, and ERS constitute the most common cardiac channelopathies associated with the risk of malignant arrhythmias occurrences, such as TdP, VT, and VF, ultimately leading to SCD. 

Various studies aim to elucidate the complicated pathophysiology of channelopathies and define factors contributing to arrhythmias. Established principles of cardiac arrhythmia formation can be subdivided into two groups: abnormal impulse formation and reentry. The role of enhanced pacemaker or parasystole in channelopathies and associated VAs is not fully explained. However, gene mutations present in channelopathies correspond with recently defined genetic abnormalities in conduction system malfunction. The accurate role of EADs, despite previous studies, is uncertain. In LQTS, one of the most extensively studied channelopathies, and among associated VA, TdP seems to be highly associated with EAD occurrence. However, the mechanism of its initiation and perpetuation may depend on reentry. In contrast, a less controversial but poorly studied mechanism of CPVT formation indicates the major role of DADs. In BrS, two theories explain the mechanism of VA, involving the role of phase 2 reentry and RVOT depolarization. It should be noted that mechanisms from both groups may co-occur and be complementary, especially among patients with IVF.

In recent years, several studies aimed to determine genes and their mutations responsible for each channelopathy. Well-established genes causing LQTS comprise *KCNQ1, KCNH2, and SCN5A*, whereas many other gene variants are claimed to be associated with LQTS occurrence. Approximately 20% of patients with BrS have a pathological variant of the *SCN5A* gene, the only gene with confirmed significance in the diagnostic process. 

**Figure 6:**
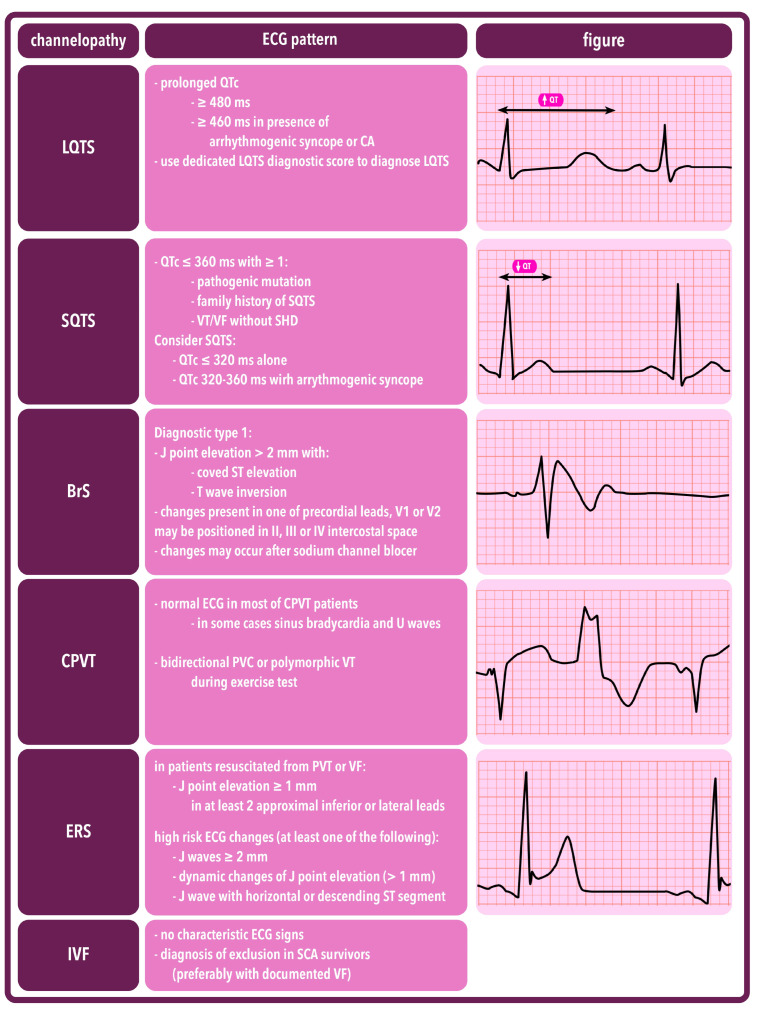
ECG patterns in channelopathies. BrS—Brugada syndrome; CA—cardiac arrest; CPVT—catecholaminergic polymorphic ventricular tachycardia; ECG—electrocardiography; ERS—early repolarization syndrome; IVF—idiopathic ventricular fibrillation; LQTS—long QT syndrome; PVC—premature ventricular complex; SCA—sudden cardiac arrest; SHD—structural heart disease; SQTS—short QT syndrome; QTc—corrected QT; VF—ventricular fibrillation; VT—ventricular tachycardia.

In many cases with a suspicion of primary electrical heart disease, the diagnostic process is complicated. Therefore, in several diseases, such as LQTS or BrS, diagnostic scores were proposed. In all of the presented diseases, 12-lead ECG has a primary role, especially at the outset of the diagnostic process. Apart from 12-lead ECG, other long-term methods should be considered, especially with regard to various factors, e.g., sympathetic activity or HR, that modify the ECG and may alter between non-diagnostic and diagnostic patterns. The most common ECG patterns are presented in Figure 6.

Finally, it should be emphasized that the thigh variety of emerging data, high complexity, and small study groups accompanied with animal study data indicate the need for further studies. An understanding of the genetic and pathophysiological aspects of channelopathies may assist the diagnostic process of primary and secondary prevention, which seems to be crucial for decreasing the global burden of SCD. It should be noted that in patients with suspected primary electrical heart disease, the current effective guidelines should be followed.

## Figures and Tables

**Table 1:** Definitions of sudden cardiac death (SCD) and sudden infant death syndrome (SIDS) [6].

	SCD	SIDS
Age	Over 1 year old	Under 1 year old
Evaluation	Unforeseen death of natural origin, with an unknown or cardiac cause	Absence of findings in pathology, toxicology, and forensic analysis
Time of death	Within one hour of symptom onset in witnessed cases and within 24 h of the last confirmed sighting in unwitnessed incidents	

SCD—sudden cardiac death; SIDS—sudden infant death syndrome.

**Table 2:** Long QT syndrome (LQTS) diagnostic criteria.

			Points
ECG findings	QTc	>480 ms	3
		460–470 ms	2
		450–459 ms	1
	TdP		1
	T wave alternans		1
	Notched T wave in 3 leads		1
	Low HR for age		0.5
Clinical history	Syncope	With stress	2
		Without stress	1
	Congenital deafness		0.5
Family history	Family members with definite LQTS1		1
	Unexplained SCD below age 30 among immediate family members		0.5

Interpretation: ≤1 point = low probability of LQTS. >1 to 3 points = intermediate probability of LQTS. ≥3.5 points = high probability of LQTS. QTc—corrected QT interval; TdP—torsade de pointes; HR—heart rate; LQTS1—long QT syndrome type 1; SCD—sudden cardiac death.

**Table 3:** Short QT syndrome subtypes [86,87,88,89].

SQTS Subtype	Gene	Channel Function	Mechanism
SQTS1	* KCNH2 *	α-subunit I_Kr_	Gain-of-function
SQTS 2	* KCNH2 *	α-subunit I_Ks_	Gain-of-function
SQTS 3	* KCNJ2 *	α-subunit I_K1_	Gain-of-function
SQTS 4	* CACNA1C *	α-subunit I_CaL_	Loss-of-function
SQTS 5	* CACNB2 *	β2-subunit I_CaL_	Loss-of-function
SQTS 6	* CACNA2D1 *	Δ1-subunit I_CaL_	Loss-of-function
SQTS 7	* SCN5A *	α-subunit I_Na_	Loss-of-function
SQTS 8	* SLC4A3 *	AE3 anion exchanger	Loss-of-function

SQTS—short QT syndrome.

**Table 4:** Hypotheses of Brugada syndrome (BrS) development [112,113].

Depolarization Disorder	Repolarization Disorder
As a result of reduced I_Na_ and interrupted conduction, delayed conduction is observed in the RVOT, especially in conjunction with the existence of late potentials. This delayed conduction leads to variable depolarization around the RVOT, which is thought to be a contributing factor in the development of arrhythmias.	A comparatively elevated outward I_to_ during the second phase of the AP, a divergence of the transatrial AP (epicardial–atrial gradient) is observed. The difference in repolarization between the epicardium and endocardium is the source of arrhythmias through phase 2 reentry. The atrial I_to_ frequency is identified as the underlying cause of atrial disease and atrial arrhythmias.

RVOT—right ventricular outflow tract, AP—action potential.

**Table 5:** Genes associated with Brugada syndrome [6,108,118,119,120,121].

Gene	Protein
*SCN5A*	Sodium channel protein type 5 subunit α
*SCN10A*	Sodium channel protein type 10 subunit α
*SCN1B*	Sodium channel subunit β-1
*SCN2B*	Sodium channel subunit β-2
*SCN3B*	Sodium channel subunit β-3
*KCND2*	Potassium voltage-gated channel subfamily D member 2
*KCND3*	Potassium voltage-gated channel subfamily D member 3
*KCNE2*	Potassium voltage-gated channel subfamily E member 2
*KCNE3*	Potassium voltage-gated channel subfamily E member 3
*KCNE5*	Potassium voltage-gated channel subfamily E regulatory β subunit 5
*KCNH2*	Potassium voltage-gated channel subfamily H member 2
*HCN4*	Potassium/sodium hyperpolarization-activated cyclic nucleotide-gated channel 4
*KCNJ8*	ATP-sensitive inward rectifier potassium channel 8
*CACNA1C*	Voltage-dependent L-type calcium channel subunit α-1C
*CACNA2D1*	Voltage-dependent calcium channel subunit α-2/δ-1
*CACNB2*	Voltage-dependent L-type calcium channel subunit β-2
*ABCC9*	ATP-binding cassette sub-family C member 9
*FGF12*	Fibroblast growth factor 12
*GPD1L*	Glycerol-3-phosphate dehydrogenase 1-like protein
*PKP2*	Plakophilin-2
*RANGRF*	Ran guanine nucleotide release factor
*SEMA3A*	Semaphorin-3A
*SLMAP*	Sarcolemmal membrane-associated protein
*TRPM4*	Transient receptor potential cation channel subfamily M member 4

**Table 6:** Shanghai Score System [122,123].

Feature	Points
1. ECG (12 lead/ambulatory)—one item from this category must apply	
(a) Spontaneous type 1 Brugada ECG pattern at nominal or high leads	3.5
(b) Fever-induced type 1 Brugada ECG pattern at nominal or high leads	3
(c) (Type 2 or 3 Brugada ECG pattern that converts with provocative drug challenge	2
2. Family history—only award points once for the highest score within this category	
(a) First- or second-degree relative with definite BrS	2
(b) Suspicious SCD (fever, nocturnal, Brugada aggravating drugs) in a first- or second-degree relative	1
(c) Unexplained SCD <45 years in first- or second-degree relative with negative autopsy	0.5
3. Clinical history—only award points once for the highest score within this category	
(a) Unexplained cardiac arrest or documented VF/polymorphic VT	3
(b) Nocturnal agonal respirations	2
(c) Suspected arrhythmic syncope	2
(d) Syncope of unclear mechanism/unclear etiology	1
(e) Atrial flutter/fibrillation in patients <30 years without alternative etiology	0.5
4. Genetic test result	
Probable pathogenic mutation in BrS susceptibility gene	0.5

Score (at least 1 ECG finding): ≥3.5 points: probable and/or definite BrS; 2–3 points: possible BrS; <2 points: non-diagnostic. BrS—Brugada syndrome; SCD—sudden cardiac death; VF—ventricular fibrillation; VT—ventricular tachycardia.

**Table 7:** Mutations and polymorphisms in ERS.

Channel	Protein	Gene	Mutation	Reference
VGKC	KCNE1	*KCNE1*	Missense p.S38G	[182]
	K_v_4.3	*KCND3*	Missense p.G306A	[170]
			Missence p.V392I	[171,172]
			Duplication	[174]
	K_v_11.1	*KCNH2/hERG*	Missense p.K801T	[181]
K_ATP_	K_ir_6.1	*KCNJ8*	Missense p.S422L	[178,179]
	SUR2A	*ABCC9*	Missense p.V734I, p.R663C, p.A665T, p.V1137I	[180]
	DPP6	*DPP6*	Missense p.L747P	[175]
VGCC	Alpha 1c subunit Ca_V_1.2	*CACNA1C*	Missense p.G490R	[189]
			Missense p.P817S	[180,189]
			Missense p.G37R	[156,157,189]
			Frameshift p.E850del	[156,189]
			Missense p.Q1916R	[155]
	Beta-2 subunit	*CACNB2*	Missense p.A170V, S503L	[189]
			Missense p.S160T, p.R571C	[156,189]
			Missense p.R552G	[156]
		*CACNB2b*	Missense p.S143F	[157]
	Alpha-2/delta-1 subunit	*CACNA2D1*		[189]
			Missense p.S956T	[156]
VGSC	Alpha subunit Na_v_1.5	*SCN5A*	Frameshift p.C280S*fs61	[183]
			Missense p.Y352C	[185]
			Missense p.G1433A	[184]
			Nonsense p.K249X, p.Q1185X, Missence p.G452C, p.V728I, p.G1408R, p.L1412F, p.S1787N, Frameshift p.Q646RfsX6, p.V845CfsX2, p.G1297GfsX22	[154]
			Missense p.E1784K	[180]
	Alpha subunit Na_v_1.8	*SCN10A*		[180]
	Beta-1 subunit	*SCN1Bβ*	Missence p.S248R p.R250T	[188]
	Plakophilin-2	*PKP*	Missence p.D26N	[187]
	GPD1-L	*GPD1-L*	Missense: p. P112L	[186]

VGCC—voltage-gated calcium channel; VGSC—voltage-gated sodium channel; VGPC—voltage-gated potassium channel.

## Data Availability

The data used in this article were sourced from materials mentioned in the References section.
